# Which Zebrafish Strains Are More Suitable to Perform Behavioral Studies? A Comprehensive Comparison by Phenomic Approach

**DOI:** 10.3390/biology9080200

**Published:** 2020-08-01

**Authors:** Gilbert Audira, Petrus Siregar, Stefan-Adrian Strungaru, Jong-Chin Huang, Chung-Der Hsiao

**Affiliations:** 1 Department of Chemistry, Chung Yuan Christian University, Chung-Li 320314, Taiwan; gilbertaudira@yahoo.com (G.A.); siregar.petrus27@gmail.com (P.S.); 2 Department of Bioscience Technology, Chung Yuan Christian University, Chung-Li 320314, Taiwan; 3 Department of Research, Faculty of Biology, “Alexandru Ioan Cuza” University of Iasi, 700505 Iasi, Romania; stefan.strungaru@uaic.ro; 4 Department of Applied Chemistry, National Pingtung University, Pingtung 900391, Taiwan; 5 Center of Nanotechnology, Chung Yuan Christian University, Chung-Li 320314, Taiwan

**Keywords:** zebrafish, strain, behavior, anxiety, aggressiveness, fear, social interaction

## Abstract

Wild-type (WT) zebrafish are commonly used in behavioral tests, however, the term WT corresponds to many different strains, such as AB, Tübingen long fin (TL), and Wild Indian Karyotype (WIK). Since these strains are widely used, there has to be at least one study to demonstrate the behavioral differences between them. In our study, six zebrafish strains were used, which are AB, absolute, TL, golden, pet store-purchased (PET), and WIK zebrafishes. The behavior of these fishes was tested in a set of behavioral tests, including novel tank, mirror-biting, predator avoidance, social interaction, and shoaling tests. From the results, the differences were observed for all behavioral tests, and each strain displayed particular behavior depending on the tests. In addition, from the heatmap and PCA (principal component analysis) results, two major clusters were displayed, separating the AB and TL zebrafishes with other strains in another cluster. Furthermore, after the coefficient of variation of each strain in every behavioral test was calculated, the AB and TL zebrafishes were found to possess a low percentage of the coefficient of variation, highlighting the strong reproducibility and the robustness of the behaviors tested in both fishes. Each zebrafish strain tested in this experiment showed specifically different behaviors from each other, thus, strain-specific zebrafish behavior should be considered when designing experiments using zebrafish behavior.

## 1. Introduction

In recent decades, molecular biology has obtained a crucial position in many research fields, including the neurobiological mechanisms underlying diseases, drug actions, and behavior. Hence, knockout and transgenic animals, such as Drosophila [1,2], fish [3,4], and rodents [5,6] became popular tools in this field. However, one has to consider the genetic background of the individuals before conducting the experiment, especially in behavioral studies, since nowadays, plentiful evidence of behavioral task differences between the strains exists [7]. In the literature focused on rodents models, a lot of inbred strains have been developed and maintained, such as the Wistar–Kyoto strains of normotensive (WKY) and spontaneously hypertensive (SHR) rats [8]. For the study of genetically derived hypertensive disease, these strains are important resources, in addition to being subjects for behavioral studies. After subsequent behavioral studies, it was found that SHR rats possessed different behaviors from their WKY controls, including increased behavioral responses to environmental stimuli, hyperactivity to stressful stimuli, and the faster acquisition of active avoidance [9,10]. Another study supported the results and it considered WKY rats as particularly underactive when compared with other genetic strains of normotensive rats [11]. In addition, another prior study in eight inbred strains (129S1/SvImJ, 129S6/SvEvTac, BALB/cByJ, A/J, C57BL/6J, C3H/HeJ, FVB/NJ, and DBA/2J) and one outbred strain (CD1-ICR) also showed significant strain differences in the light–dark exploration test [12]. Furthermore, another mice behavioral study discovered that T1 and DBA/2 male mice react very differently to prior novelty experience and revealed a significant strain difference in plus-maze profiles [13].

In recent years, a significant escalation in the use of adult zebrafish (*Danio rerio*) has been seen in behavioral neuroscience research [14]. Offering several advantages including low husbandry costs, easy maintenance, and small size, these freshwater fish have become an excellent model organism for effective experimental use [15,16]. Furthermore, because of their vast use in research, some inbred zebrafish strains are available, differing not only morphologically, but also physiologically, genetically, and behaviorally, in both the larval and adult stages, and these differences may cause differences in the behavioral test outcomes [17,18]. However, while one can have vast knowledge about the feature of different outbred and inbred strains in rodents, only a few studies focused on strain variation in zebrafish, especially in their behavior [1]. Nevertheless, a prior study has reported behavioral differences between Tübingen (TU), AB, Wild Indian Karyotype (WIK), and shortfin (SF) zebrafish after ethanol exposure, while others have discovered differences between AB and TU zebrafish in a light–dark preference test, color conditioning, and exploratory behavior [19,20]. In addition, the less anxious behavior of wild-type zebrafish, including leopard, long-fin, and albino zebrafish strains in a novel tank test were demonstrated by Egan and colleagues [21]. Differences in a novel tank test and shoaling behavior between WIK and AB zebrafish were also shown from other previous studies [22,23]. Taken together, a better understanding of zebrafish strain-specific behavior variation can help in the selection of an appropriate assay in relation to the strains that are used.

In the current study, we followed up on these potential behavioral findings by further characterizing several zebrafish strains, which are AB, absolute, Tübingen long fin (TL), golden, pet store-purchased (PET), and WIK (Wild Indian Karyotype), with the comprehensive well established behavior assays that were established in our previous publication, including novel tank, mirror-biting, predator avoidance, social interaction, and shoaling tests [24]. In addition, to conduct a deeper study about their behavior differences, Tiger barb (*Puntigrus tetrazona*) was used as the outgroup. We hope these findings can help other researchers to identify which strain is more suitable for the behavioral test run in the future, mainly in an ecotoxicology context, and contribute to the growing database of phenotypical differences between several common strains of zebrafish.

## 2. Materials and Methods

### 2.1. Zebrafish Strains Used in This Study

The AB, TL (Tübingen long fin), WIK (Wild Indian Karyotype), and absolute mutants originated from Taiwan Zebrafish Core Facility at Academia Sinica (http://icob.sinica.edu.tw/tzcas/). TL fish is a double mutant line with a recessive pigment (*leo^t1^*) and a dominant long-fin (*lof^dt2^*) mutation [25]. *Leo^t1^* is a recessive mutation causing spotting in adult fish, while the long fish are caused by *lof^dt2^*, a dominant, homozygous viable mutation [26]. Absolute mutant fish is a double mutant fish carrying *ednrb1a^b140^* and *mitfa^b692^* and lacks melanophore, xantophore, and most iridophore cells, causing a transparent body appearance [25]. WIK zebrafish were originally derived from a single pair mating of second-generation wild-caught Indian zebrafish (WIK11) in the mid-1990s. Since the WIK line is highly polymorphic in comparison to the TU (Tübingen) and AB line, it is commonly used for genome mapping [1,27]. The golden zebrafish were obtained from the pet store or local aquarium, as well as the PET zebrafish used in this experiment. The golden zebrafish identified as the zebrafish with a mutation in *slc24a5*, resulting in a lightening of the pigmented lateral stripes in adult fish, caused by a delay in melanin deposition during embryogenesis, and the production of small and irregular-shaped melanin granules [28,29], while PET zebrafish represent wild-type genetically undefined and also genetically heterogeneous stock [30]. In addition, Tiger barb (*Puntigrus tetrazona*) was also purchased from the local breeders. All the fish in each experiment were 6–12 month-old adults with a total number of 202 (*n* = 30 AB; *n =* 30 TL; *n* = 23 WIK; *n* = 30 absolute mutants; *n* = 30 golden; *n* = 30 PET; *n* = 29 *P. tetrazona*). The mixed gender of the zebrafish in each group was used in the present study. The AB strain had been reared in the zebrafish facility for more than twenty generations. In the zebrafish facility, the AB strain zebrafish were bred, raised, and maintained in a recirculating aquatic system with a 10/14 h dark/light cycle at 28 ± 1 °C, according to the standards [31]. Circulating water in the aquarium with the conductivity of the system water was kept between 300 and ~1500 µS and was constantly filtered by the ultraviolet (UV) light and had a pH 7.0–7.5. The zebrafish were fed twice a day with either commercial dry food or lab-grown brine shrimp. Then, even though the other fish (*absolute*, TL, *golden*, PET, WIK zebrafishes and tiger barb fish) originated from other facilities with similar housing and maintenance conditions, these fishes were still reared in the zebrafish facility for at least 1 month prior to the experiment. This process was necessary to eliminate the external factors, such as the stressful condition during the transfer, that might affect their behavior performance without disregarding the readily acclimatizing to the new environment trait of zebrafish [32]. The general maintenance procedures and housing conditions were as previously described by Avdesh et al. 2012 [31].

### 2.2. Zebrafish Behavioral Tests and Ethics

All the animal experiments were performed in accordance with the guidelines issued by the Institutional Animal Care and Use Committees (IACUCs) of Chung Yuan Christian University (application number: CYCU106025, issue date 6 May 2018). After acclimation in the zebrafish facility, a set of behavioral tests, including novel tank exploration, mirror-biting, predator avoidance behavior, conspecific social interaction, and shoaling tests were conducted in the entire fish group within the morning until afternoon (10:00 to 16:00) after the routine morning feeding either with commercial dry food or brine shrimp [24]. The whole behavioral test was conducted in a room with 25 ± 1 °C temperature. A trapezoid tank with 28 cm at the top, 22 cm along the bottom, 15.9 cm along the diagonal side, and 15.2 cm high, filled with 1.25 L of water, was used for the tested fish, individually, in all of the behavior tests, except in the shoaling test (shoal size = 3). After 5 min of acclimation in the tank, except for the novel tank test, the fish behaviors were recorded after being introduced to the specific stimulus (the image reflection in the mirror-biting test, the *Amatitlania nigrofasciata* in the predator avoidance test, and the conspecific presence in the social interaction test). The recording process lasted for 5 min for each behavioral test, excluding the novel tank test, which was 1 min for every 5 min of the test in a 30 min time interval. Afterward, the videos were analyzed by idTracker based on the previous method [24].

### 2.3. Video-Tracking and Statistical Data Analysis

A desktop computer (Intel i7-5820K core @ 3.3 GHz and 64 GB of RAM) was used to process all the zebrafish behavior videos. Subsequently, idTracker (http://www.idtracker.es/) open source software was used to collect and convert the zebrafish movement data into trajectories, as previously described [24,33]. Movement tracking was carried out separately for each tank. Later, all of the behavioral data were analyzed and plotted by using GraphPad Prism (GraphPad Software version 8 Inc., La Jolla, CA, USA), a scientific graphing and statistics software, except for the novel tank test result. The novel tank test results and locomotor trajectories for each test were plotted by using Origin^®^ 2019b (Origin^®^ Software version 2019b Inc., Northampton, MA, USA). For the data analysis, every fish group was individually compared to the AB strain zebrafish group, utilizing either the Mann–Whitney test, a test to find that the difference between the distribution of ranks among the two groups is statistically significant, or two-way ANOVA, a test to determine how a response is affected by two factors, with the Geisser–Greenhouse correction, since the behavioral data did not normally distribute [34]. The AB strain zebrafish group was used as the comparison because this strain is a widely used strain of zebrafish [35,36]. Data for each zebrafish fish group were expressed as median with interquartile range.

### 2.4. Principal Component Analysis (PCA), Heatmap, and Hierarchical Clustering Analysis

All of the behavioral endpoint’s values from all of the tested zebrafish in each behavior test were inserted into an excel file using Microsoft Excel and afterward, the excel file was converted into a comma-separated values type file (.csv). The definition of all of the behavioral endpoints was described in Supplementary Table S1 and more detailed descriptions for each test were explained in the prior study [24]. Later, the file was uploaded to a web tool designed for clustering and visualizing multivariate data, ClustVis (https://biit.cs.ut.ee/clustvis). Then, to treat each variable equally, unit variance scaling for each row was applied. Since there were no missing values in the dataset, singular value decomposition (SVD) with the imputation method was utilized to calculate the principal components as described by the previously published method [37]. Finally, after the data processing, the results of the PCA and heatmap were exported and saved in the system. The color bar beside the heatmap clustering showed the range of the behavioral endpoint value after data calculation. Low values tended towards a blue color tone while the higher values tended towards a red color tone.

## 3. Results

### 3.1. Absolute, Golden, and Pet Store-Purchased (PET) Zebrafish Displayed Different Behaviors in the Novel Tank Test

**Figure 1:**
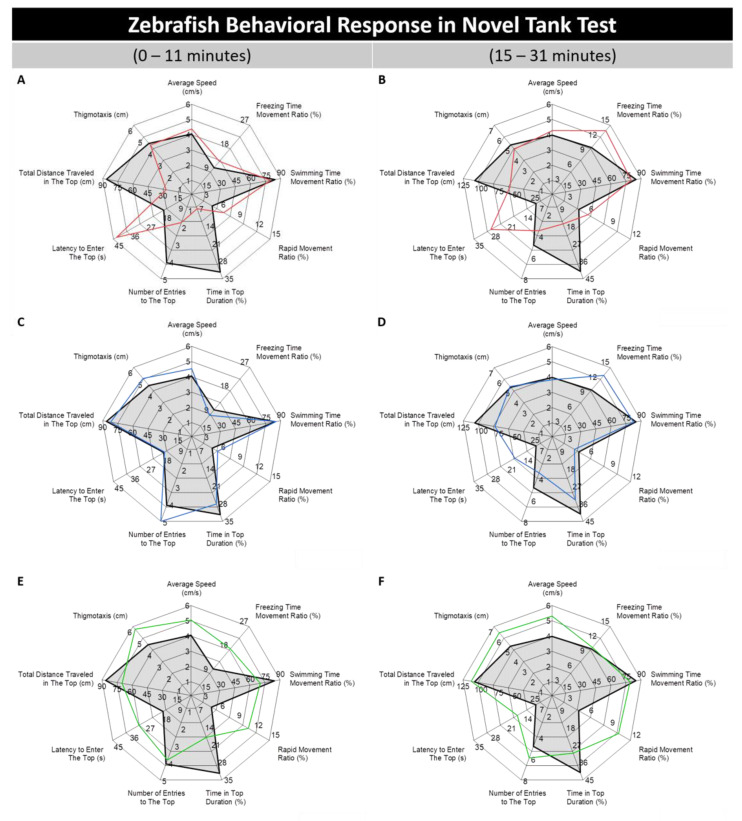

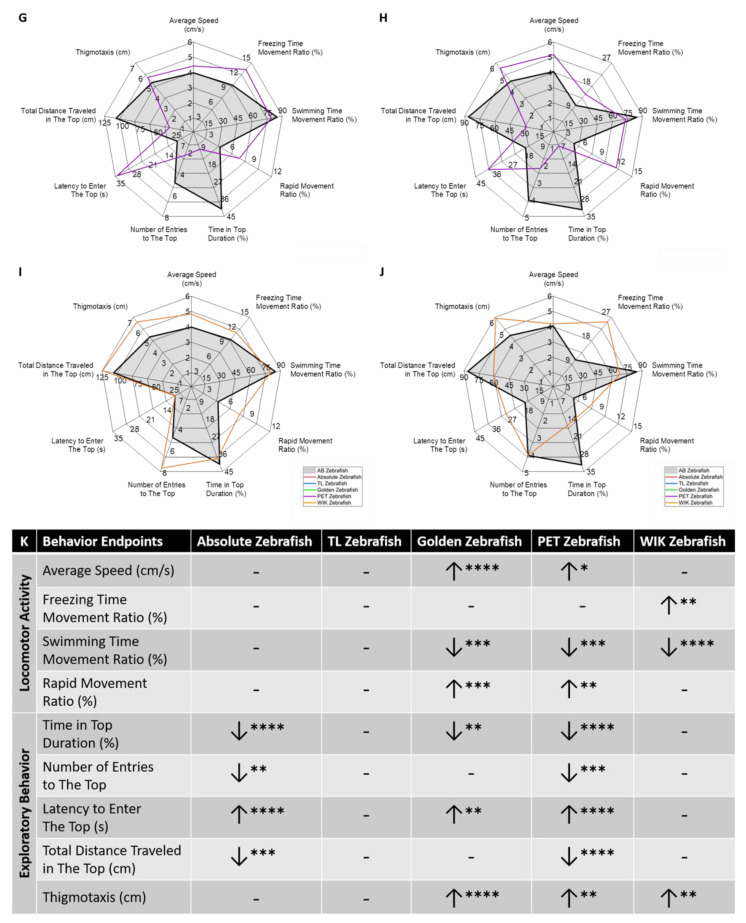
Behavioral response profile plots of AB zebrafish (black) compared to the (**A**,**B**) absolute mutant zebrafish (red), (**C**,**D**) Tübingen long fin (TL) zebrafish (blue), (**E**,**F**) golden zebrafish (green), (**G**,**H**) pet store-purchased (PET) zebrafish (purple), and (**I**,**J**) Wild Indian Karyotype (WIK) zebrafish (orange) before and after 11 min of exposure of a new environment in the novel tank test, respectively. The plots are expressed as the mean and all of the fish swimming activities during a 31 min test with a 1 min video with a time interval every 5 min were summarized and analyzed using two-way ANOVA with the Geisser–Greenhouse correction. (**K**) Summary table of the behavioral differences of several strains of zebrafish compared to the AB strain zebrafish. ↑ represents a significant increase in the value and ↓ indicates a significant decrease in the value in comparison to the AB strain zebrafish group, respectively, while “-” represents a non-statistically different in the value in comparison to the AB strain zebrafish group (*n* = 30 for each group, except for the WIK zebrafish (*n* = 23); * *p*< 0.05; ** *p* < 0.01; *** *p*< 0.001; **** *p*< 0.0001).

The novel tank test is an assay to examine the fish’s adaptation ability in a novel environment by exploiting the tendency of zebrafish to display a high-anxiety level with typical bottom-dwelling behavior during the first several minutes and exploration into the upper arena, followed with a reduction of their anxiety level, gradually [38]. After the test was conducted, the behavior response variables were structured to include the average speed, freezing, swimming, and rapid movement ratios, time in top duration, number of entries to the top, latency to enter the top, total distance traveled in the top, and thigmotaxis in different regions of each plot. Two radar plots were built to distinguish the zebrafish behavior differences before and after acclimation, which normally occurs during the first 10 min of novel tank exposure. From the two-way ANOVA result, hyperactivity-like behavior was displayed by golden and PET zebrafish, which were supported by a high level of average speed (*p* < 0.001; F (1, 58) = 13.7, *p* < 0.05; F (1, 58) = 5.36, respectively) and rapid movement ratio (*p* < 0.001; F (1, 58) = 15.2, *p* < 0.01; F (1, 58) = 9.13, respectively) (Figure 1E–H). A reduced swimming time movement ratio was also displayed by these groups (*p* < 0.001; F (1, 58) = 12.7, *p* < 0.001; F (1, 58) = 16.5, respectively). (Figure 1K). However, higher locomotor activity than the PET zebrafish was shown by golden zebrafish. This phenomenon is supported by a high level of average speed possessed by the golden zebrafish group constantly during the whole 31 min test that was not shown by the PET zebrafish after 15 min of exposure of a novel tank (Figure 1E–H). Interestingly, even though their locomotor activity was not significantly different from AB zebrafish, a significantly different movement type was shown by the WIK fish, supported by the high level of freezing time movement ratio (*p* < 0.01; F (1, 51) = 8.14) with a low level of swimming time movement ratio (*p* < 0.001; F (1, 51) = 22.1) (Figure 1I,J). Subsequently, it was also found that the absolute, golden, and PET zebrafish displayed abnormal exploratory behavior during the test. This alteration was supported by reduced time in the top duration (*p* < 0.001; F (1, 58) = 40, *p* < 0.01; F (1, 58) = 7.32, *p* < 0.001; F (1, 58) = 61.4, respectively), the number of entries to the top (*p* < 0.01; F (1, 58) = 7.34, *p* < 0.001; F (1, 58) = 14.9, respectively), the total distance traveled in the top (*p* < 0.001; F (1, 58) = 15.8, *p* < 0.001; F (1, 58) = 38.5, respectively), the high level of latency to enter the top (*p* < 0.001; F (1, 58) = 51.7, *p* < 0.01; F (1, 58) = 9.32, *p* < 0.001; F (1, 58) = 58.8, respectively) and the average distance to the center of the tank (thigmotaxis) (*p* < 0.001; F (1, 58) = 22.9, *p* < 0.01; F (1, 58) = 8.02, respectively) (Figure 1A,B,E–H). In addition, an elevated thigmotaxis level (*p* < 0.01; F (1, 51) = 12.1) was also displayed by WIK zebrafish. Nevertheless, even though these four groups shared similar behavior alterations in terms of their exploratory behavior, the magnitude of the abnormality varied between groups. The degree of the differences was ranked from the most dissimilar to the most similar with the AB strain zebrafish as it follows the PET zebrafish, absolute zebrafish, golden zebrafish, and WIK zebrafish (Figure 1A–J). This result is deduced since in the PET zebrafish group, all of the exploratory behavior-related endpoints were found to be significantly different from those from AB strain zebrafish while it was only four, three, and one abnormal behavior-related endpoints were detected in absolute, golden, and WIK zebrafish groups, respectively (Figure 1K). On the contrary, similar behavior to the AB strain zebrafish regarding the response to the novel environment was displayed by the TL zebrafish (Figure 1C,D,K). The raw data of the novel tank test result can be found in Supplementary Table S2.

### 3.2. Different Level of Aggressiveness from AB Zebrafish Was Displayed by Absolute, Tübingen Long Fin (TL), Golden, and Wild Indian Karyotype (WIK) Zebrafish

**Figure 2:**
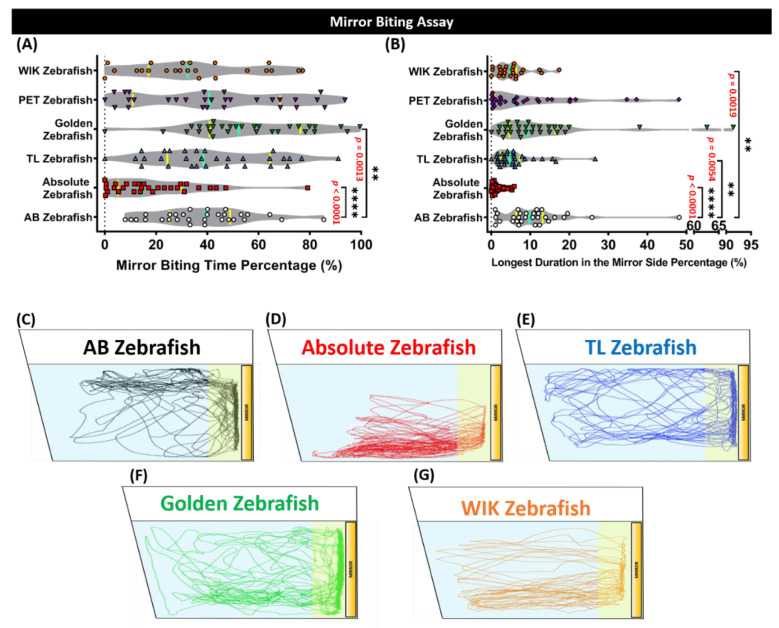
Mirror-biting behavior endpoint comparisons between the AB zebrafish (white), absolute zebrafish (red), TL (Tübingen Long Fin) zebrafish (blue), golden zebrafish (green), PET (Pet Store-purchased) zebrafish (purple), and the WIK (Wild Indian Karyotype) zebrafish (orange). (**A**) Mirror-biting time percentage and (**B**) the longest duration in the mirror side percentage were analyzed for the mirror-biting assay. The median and the interquartile for the violin plot were labeled with the bold line colored with cyan and yellow, respectively. The 5 min locomotor trajectories for the AB, absolute, TL, golden, and the WIK zebrafishes are presented in (**C**–**G**), respectively, with the yellow-colored zone as the mirror-biting region. The data were analyzed by a Mann–Whitney test (*n* = 30 for each group, except for the WIK zebrafish (*n* = 23); ** *p* < 0.01; **** *p* < 0.0001).

To evaluate fish aggressiveness, the mirror-biting assay was conducted. This simple and efficient method is used to measure the level of aggressiveness in zebrafish by monitoring the frequency of the zebrafish to bite their image reflection in the mirror [39]. In addition, this test may also indicate the intent or social motivation of zebrafish to interact with a social partner [40]. From the result, a low level of aggressiveness was exhibited by the absolute, TL, and WIK zebrafish groups. However, among these groups, the lowest level of aggressiveness was shown by the absolute strain, indicated by a significant reduction of mirror-biting time percentage (*p* < 0.001) and the longest duration in the mirror side percentage (*p* < 0.001) endpoints compared to the AB strain, confirmed by the Mann–Whitney test. On the other hand, only the longest duration in the mirror side percentage endpoint was found to be significantly lower in the TL (*p* < 0.01) and WIK (*p* < 0.01) strains (Figure 2A,B). Interestingly, a slightly high level of aggressiveness was displayed by the golden strain (*p* < 0.01), shown by a high level of mirror-biting time percentage (Figure 2A). Besides, a similar level of aggressiveness with the AB strain zebrafish was observed in the PET zebrafish (Figure 2A,B). Taken together, all of the zebrafish strains tested in this experiment showed different levels of aggressiveness. Figure 2C–G display the locomotor trajectories for the AB, absolute, TL, golden, and WIK zebrafishes in the mirror-biting test.

### 3.3. Absolute, Tübingen Long Fin (TL), and Wild Indian Karyotype (WIK) Zebrafish Displayed Less Predator Avoidance Behaviors than the AB Zebrafish in the Predator Avoidance Test

**Figure 3:**
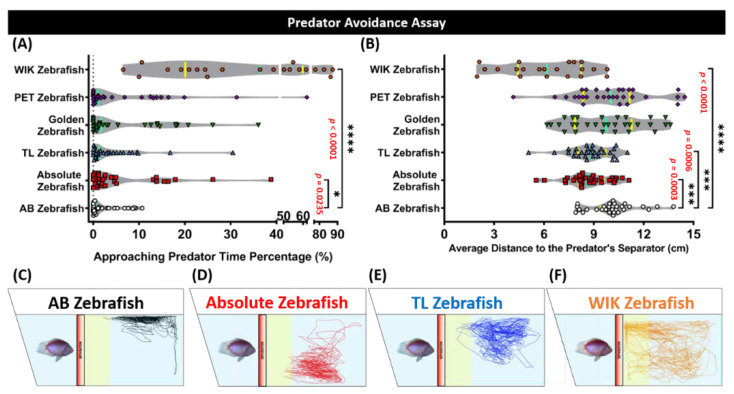
Predator avoidance behavior endpoint comparisons between the AB zebrafish (white), absolute zebrafish (red), TL (Tübingen Long Fin) zebrafish (blue), golden zebrafish (green), PET (Pet Store-purchased) zebrafish (purple), and WIK (Wild Indian Karyotype) zebrafish (orange). (**A**) Approaching the predator time percentage and (**B**) the average distance to the predator’s separator were analyzed for the predator avoidance assay. The median and the interquartile for the violin plot were labeled with the bold line colored with cyan and yellow, respectively. The 5-min locomotor trajectories for the AB, absolute, TL, and WIK zebrafishes are presented in (**C**–**F**), respectively, with the yellow colored zone as the approaching predator region. The data were analyzed by a Mann–Whitney test (*n* = 30 for each group, except for the WIK zebrafish (*n* = 21); * *p* < 0.05; *** *p* < 0.001; **** *p* < 0.0001).

Generally, when encountering their natural predator, fish will show freezing behavior or high anxiety as their innate response. Similarly, zebrafish also possess this predator avoidance behavior and this response is useful in examining certain behavior alterations during the course of revelation to the predator [41]. Based on a previous publication [24], convict cichlid fish (*Amatitlania nigrofasciata*) were used in this experiment as the stimulus to observe the tested fish predator avoidance behavior. The Mann–Whitney test revealed that the absolute, TL, and the WIK strains displayed significantly different predator avoidance behaviors compared to their AB strain counterpart. However, the WIK strain displayed the most different response to the predator presence. This phenomenon was shown by the highest level of approaching predator time percentage (*p* < 0.001) and the lowest level of the average distance to the predator’s separator (*p* < 0.001) observed in this group after the test. Similar to this result, abnormal predator avoidance behavior was also shown by the absolute strain, which was indicated by significantly different levels of both endpoints (*p* < 0.05, *p* < 0.001) even though not as different as the WIK strain. Based on this result, locomotor activity for each group was measured since they are distinct from normal swimming behavior and might be related to the fear response of the zebrafish. After further examination of their locomotor activity during the test, it was found that the WIK and absolute strains exhibited abnormal locomotion compared to the other groups. This abnormality in the WIK strain was shown by a high level of average speed, swimming time movement ratio, and low level of freezing time movement ratio while in the absolute zebrafish, by a the low level of freezing, rapid movement ratios and an elevated freezing time movement ratio (Supplementary Figure S1). On the other hand, even though there were slight differences in their locomotion, the TL strain showed more similarity with the AB strain regarding the predator avoidance behavior compared to the absolute and WIK strains since only a low level of the average distance to the predator’s separator (*p* < 0.001) was observed in this group (Figure 3A,B and Supplementary Figure S1). Furthermore, no significant differences were detected in both endpoints between the AB, golden, and PET strains, except in some locomotor activity endpoints for the PET strains (Figure 3A,B and Supplementary Figure S1). The locomotor trajectories of the AB strain, absolute, TL, and WIK strains in the predator avoidance assay were summarized in Figure 3C–F.

### 3.4. Absolute, Pet Store-Purchased (PET), and Wild Indian Karyotype (WIK) Zebrafish Displayed Less Interest to the Conspecific than the AB Zebrafish in the Social Interaction Test

**Figure 4:**
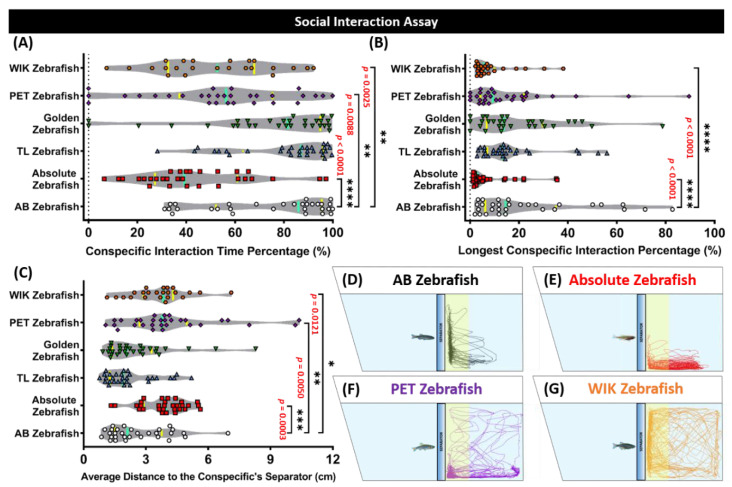
Social interaction behavior endpoint comparisons between the AB zebrafish (white), absolute zebrafish (red), TL (Tübingen Long Fin) zebrafish (blue), golden zebrafish (green), PET (Pet Store-purchased) zebrafish (purple), and the WIK (Wild Indian Karyotype) zebrafish (orange). (**A**) Conspecific interaction time percentage, (**B**) longest conspecific interaction percentage, and (**C**) the average distance to the conspecific’s separator were analyzed for the social interaction assay. The median and the interquartile for the violin plot were labeled with the bold line colored with cyan and yellow, respectively. The 5 min locomotor trajectories for the AB, absolute, PET, and WIK zebrafish are presented in (**D**–**G**), respectively, with the yellow colored zone as the interacting conspecific region. The data were analyzed by a Mann–Whitney test (*n* = 30 for each group, except for the WIK zebrafish (*n* = 23); * *p* < 0.05; ** *p* < 0.01; *** *p* < 0.001; **** *p* < 0.0001).

Then, to examine the social behavior of fish, a conspecific social interaction test was conducted. Based on a similar rodent paradigm, this test is another efficient assay to evaluate zebrafish social phenotypes by observing their interactions with the conspecifics [39]. The Mann–Whitney test results found that the absolute, PET, and WIK strains were less interested in their conspecifics than the AB strain, and exhibited a distinctive social withdrawal phenotype, which was supported by a lower conspecific interaction time percentage *p* < 0.001, *p* < 0.01, *p* < 0.01, respectively) and a higher average distance to the conspecifics separator (*p* < 0.001, *p* < 0.01, *p* < 0.05, respectively) (Figure 4A,C). In addition, a low level of the longest conspecific interaction percentage was displayed by the absolute (*p* < 0.001) and WIK strains (*p* < 0.01), emphasizing the higher magnitude of absolute zebrafish differences regarding their social interaction behavior compared to the PET zebrafish (Figure 4B). However, similar conspecific interest was observed in the TL and golden strains when the AB strain was compared (Figure 4A–C). The locomotor trajectories of the AB, absolute, PET, and WIK strains in the conspecific social interaction assay were summarized in Figure 4D–G.

### 3.5. Loose Shoal Was Formed by Pet Store-Purchased (PET) and Wild Indian Karyotype (WIK) Zebrafishes in the Shoaling Test

**Figure 5:**
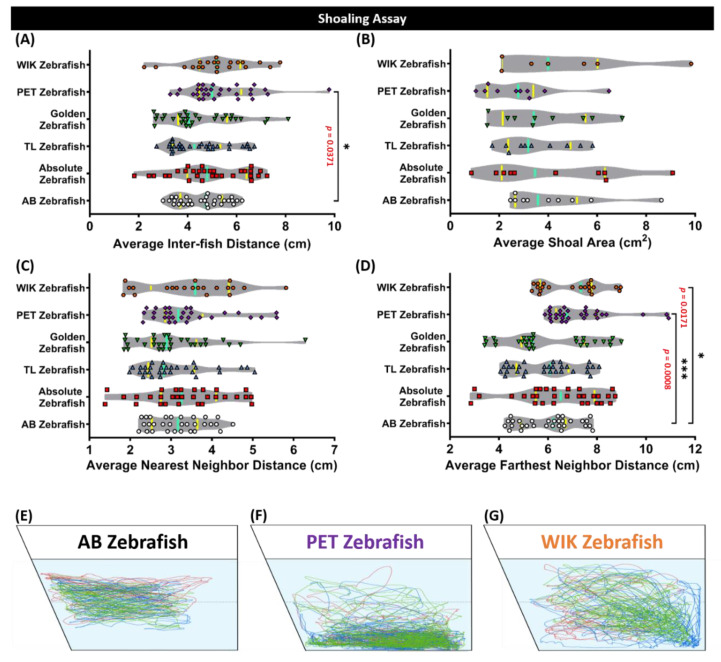
Shoaling endpoint comparisons between the AB strain zebrafish (white), absolute zebrafish (red), TL (Tübingen Long Fin) zebrafish (blue), golden zebrafish (green), PET (Pet Store-purchased) zebrafish (purple), and the WIK (Wild Indian Karyotype) zebrafish (orange). (**A**) Average inter-fish distance, (**B**) average shoal area, (**C**) average nearest neighbor distance, and (**D**) the average furthest neighbor distance were analyzed for the shoaling assay. Groups of three fish were tested for shoaling behavior. The median and the interquartile for the violin plot were labeled with the bold line colored with cyan and yellow, respectively. The 5 min locomotor trajectories for the AB, PET, and WIK zebrafish are presented in (**E**–**G**), respectively. The faint grey line indicates the borderline between the top and bottom portions of the test tank. The data were analyzed by a Mann–Whitney test (*n* = 30 for each group, except for the WIK zebrafish (*n* = 21); * *p* < 0.05; *** *p* < 0.001).

Generally, zebrafish tend to swim together as a very tight group when they sense threats, known to us as shoaling [42,43]. Shoaling is an innate social behavior for zebrafish to swim together to reduce anxiety and the risk of being captured by predators [39]. According to our endpoint analysis by the Mann–Whitney test, a loose shoal was formed by the PET strain. This phenomenon was indicated by a higher level of their average inter-fish distance (*p* < 0.05) and average furthest neighbor distance (*p* < 0.001) compared to the AB strain (Figure 5A,D), even though there was no significant difference observed in the average shoal area (*p* > 0.05) and the average nearest neighbor distance (*p* > 0.05) endpoints (Figure 5B,C). In addition, a slightly loose shoal was also formed by the WIK strain, even though it was not as loose as the PET zebrafish one, which was supported by a significantly low average furthest neighbor distance (*p* < 0.05) only (Figure 5D). On the contrary, considerable similar shoaling behavior with the AB strain was exhibited by the other strains of absolute, TL, and golden (Figure 5A–D). The locomotor trajectories of the AB, PET, and WIK zebrafishes in the shoaling assay are presented in Figure 5E–G.

### 3.6. Principal Component Analysis (PCA) Analysis and Hierarchical Clustering Analysis of Several Fish Behavioral Endpoints

**Figure 6:**
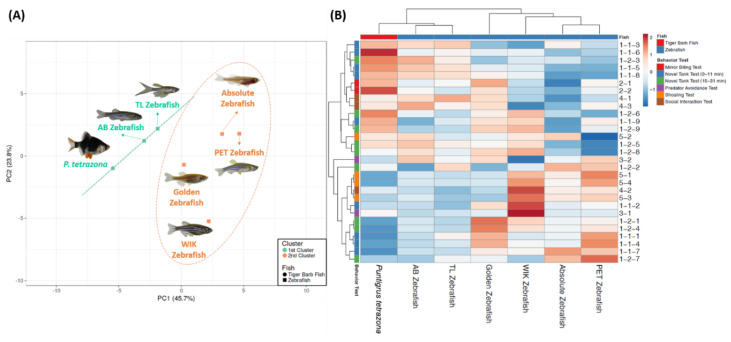
(**A**) Principal component analysis and (**B**) the hierarchical clustering analysis of the multiple behavior endpoints in several different strains of zebrafish. In Figure 6A, two major clusters from the hierarchical clustering analysis results were marked with the spring green (1st cluster) and orange (2nd cluster) circles (TL = Tübingen Long Fin, PET = Pet Store-purchased, WIK = Wild Indian Karyotype). In addition, the behavioral data from another fish species tiger barb (*Puntigrus tetrazona*) were also included as the outgroup, to conduct a deeper study about their behavior differences pattern.

After all of the fish behavioral tests, principal component analysis (PCA), heatmap, and hierarchical clustering comparison were performed in order to reduce the data dimension and complexity, and to explore the behavioral phenomics between the several different strains of zebrafish and to reconfirm the observed correlations between the experimental manipulations and behavioral endpoints. Furthermore, tiger barb (*Puntigrus tetrazona*) behavioral data were also included as the outgroup to conduct a deeper study about the behavioral pattern differences between each tested zebrafish strain. The raw data for the tiger barb fish in each behavior test can be found in Supplementary Table S3. PCA is a mathematic method to linearly reduce the data dimension and complexity, while in the heatmap clustering, the algorithm compared both columns and rows so that similar columns, and similar rows, were grouped together, with their similarity represented by a dendrogram. The PCA analysis showing all the tested fish strains can be classified into two major groups (Figure 6A). Both of the AB and TL strains were classified together in the same major cluster (first cluster, green color), leaving the rest of the fish in another major cluster (second cluster, orange color), which consists of the golden, WIK, absolute, and the PET strains. Based on the PCA phenomic analysis, we concluded that the overall behavioral performance for TL was more similar to AB strains.

Then, we performed two-dimensional heatmap hierarchy clustering to compare each behavioral endpoint among the diverse zebrafish strains. Heatmap hierarchy clustering also generated two major clusters (Figure 6B). As we expected, both of the AB and TL strains were classified together in the same major cluster (first cluster), leaving the rest of the fish in another major cluster (second cluster), which consists of the golden, WIK, absolute, and PET strains. This categorization was plausible since based on the rows clustering, behavior variables were vertically clustered in two distinct clusters, separating these two major clusters. In the first row cluster, a significant difference pattern between each major cluster was observed. A relatively high level of behavior endpoints was reported in the first cluster (red color), whereas in another major cluster, a relatively low level (blue color) was reported. After further investigation, we found that most of these differences were related to the differences in their exploratory behavior, aggressiveness, and conspecific social interaction-related endpoints. On the contrary, in the last row cluster, a relatively low level of behavior endpoints was reported in the first cluster (blue color) while a relatively high level of behavior endpoints (red color) was observed in the second cluster. Later, we found that these differences mostly occurred in the locomotor activity and shoaling-related endpoints, which were found to be significantly altered, especially in the PET strain. Interestingly, we found that the tiger barb fish were also arranged in the first cluster, thus, it can be concluded that this fish has a quite similar pattern regarding its behaviors, especially the fish behaviors examined in this experiment, with the AB and TL strains.

## 4. Discussions and Conclusions

Nowadays, since the usage of zebrafish is increasing in various fields of research, particularly in behavioral assays, the detailed knowledge of elements that can be a source of difference between replicates is needed. Several prior studies using laboratory strains and wild-type (WT) populations have shown that behavioral responses vary according to the strain used [15,20,27,44]. However, whether the fish strain was an important source of variation between the assays remains unknown [19]. Therefore, we conducted a comprehensive comparison of the behavior of several widely used laboratory zebrafish strains. In the present study, the behaviors for multiple zebrafish strains were observed and quantified by using a specially designed, the versatile function instrument that was able to perform a novel tank, mirror-biting, predator avoidance, conspecific social interaction, and shoaling tests [24]. In addition, by conducting biological repeats with high sample numbers (up to 30 individuals) can also significantly improve the statistical significance. To our knowledge, this paper is the first one attempting to perform multiple inter-strain behavioral comparisons by using up to six different zebrafish strains. Based on the results, the current study provided solid evidence to show significant inter-strain variations in each zebrafish behavioral endpoints.

In the novel tank test, higher locomotor activities than the AB strain were displayed by the golden and PET strains. It is well known that besides hypoactivity, hyperactivity is linked to the novelty of the environment [45]. Normally, as fish gradually acclimate to the new environment, adaptability in locomotor activity and exploration occurs [46]. However, this behavior was not observed in the golden zebrafish group, which may be explained by prior studies that found the differences of anxiolytic-sensitive responses to novel environments among AB, WIK, and outbred zebrafish with pigment mutations [21,22]. This different response may also elucidate the altered exploratory behavior displayed by the fish during the test. Subsequently, regarding the PET strain behavior, higher locomotor activity than the AB zebrafish was also displayed by the zebrafish from a pet store in the plus-maze center, which is consistent with the current result [23]. In addition, there is one possible explanation for the variation in the behavior observed between the PET and AB strains, which is that the PET fish reflect a wild behavioral phenotype relative to the domesticated phenotype of the AB strain. There is a line of support for this assertion. Since the PET zebrafish were obtained from the local pet store, there is a possibility that this fish was only a few generations removed from wild-caught fish and not yet evolved in response to the laboratory environment. Several studies have reported behavioral differences between wild-caught and laboratory strain populations, such as differences in behavioral activities, thigmotaxis, and social behavior [19]. Besides, the fact that in most cases of fish domestication, food is delivered to the surface of the water. Thus, domesticated fish with a greater tendency to occupy the top portions of the water tank would display more robust exploratory behavior, which could not be seen clearly in the PET zebrafish group [40,47]. Furthermore, a slightly different exploratory behavior with the AB strain was also observed in the WIK strain. After further investigation, the difference was observed in their thigmotaxis behavior. Thigmotaxis, one of the primary measures of anxiety in this model, refers to a “centrophobic” behavior, in which animals spend the majority of their time by staying near the walls of the tank [48]. Based on the result, the WIK zebrafish displayed a high level of thigmotaxis which is reasonable because the WIK fish was found to be a highly anxious zebrafish in a prior study [49]. In addition, consistently with the previous study, the WIK fish exhibited no bottom-dwelling behavior during the test [48].

Then, a mirror-biting test, which represented another modification of the novel tank test and also relevant to social behavior, was conducted. Traditionally, this test is used for studying zebrafish aggressive and social behavior by exploiting the mirror image stimulation [50]. Based on the results, several zebrafish strains displayed a significantly different level of aggressiveness than the AB strain, including the TL and WIK strains. In the TL, this phenomenon may be related to their lower cortisol levels and expression levels of the stress-axis related genes than the AB fish after the learning task, as mentioned in the previous study [20]. As it is already well known, serotonin, a steroid hormone, is secreted by the teleost fish adrenal system in response to acute and chronic stress and different levels of cortisol might contribute to the different behavior observed in this study [51]. In addition, another prior experiment observed the difference in the baseline activity of the hypothalamic–pituitary–interrenal (HPI) axis between the AB and TL larvae [52]. This finding may also help to elucidate this phenomenon since the HPI axis, which is homologous to the mammalian hypothalamic–pituitary–adrenal (HPA) axis, and behaviors are modulated in the presence of acute stress, including social stress [53]. Social stress results in general behavioral inhibition, such as decreases in aggressive social interactions and appetite [54]. In WIK strain, the slightly low level of aggressiveness might be related to the novel tank test result. As it is already mentioned above, the WIK strain displayed an obvious thigmotaxis, a sign of anxiety, and a lower level of the activity level of the zebrafish in the central area of the test tank can be considered as a decreased boldness, which is related to the aggression phenotype [55,56].

Fear responses are important behavioral reactions that have a significant fitness component. In this experiment, zebrafish fear behavior in the presence of a natural stressor was assessed by conducting a predator avoidance test [41]. During the test, the avoidance or willingness to approach the predator was then measured and the behavioral response to the stimuli can provide one measure of fear or anxiety [38,57] In agreement with a prior study in AB and TL strain anxiety-related and fear-related behaviors, the TL strain showed a slightly different predator avoidance behavior with the AB strain during the test. In their study, the TL strain was found to be easier in learning to avoid the dark compartment in a light–dark box while the adult AB strain did not [20]. As already mentioned above, this difference may be attributed to the higher baseline levels of whole-body cortisol in the AB strain, which may negatively affect the hippocampal-driven context learning [58,59]. Then, the preference of the side of the tank where the convict cichlid was shown was exhibited by the WIK strain. In line with this finding, a previous study that used the animated image of the Indian leaf fish found a similar result regarding this different behavior with the AB strain. Even though it is speculative, this finding might be explained by their potential genetic differences in their response to predators, considering that WIK is a highly polymorphic strain and genetic heterogeneity might lead to alterations in the maturity of the central nervous system, musculature, and appendages that can lead to behavioral differences [19,60,61]. In addition, based on the prior studies, this predator preference shown by the WIK fish may reflect a predator inspection behavior, an exploration behavior to the fish predator by prey fish that have shown under certain circumstances [62]. This behavior manifests as an approach of the predator and has been argued to potentially serve important adaptive functions since the prey fish can avoid performing time-consuming and energetically costly anti-predatory responses in the case of a satiated predator after inspection [63,64].

In nature as well as in the laboratory conditions, zebrafish form shoals, a behavior in which individual members swim close to each other, forming a group, under stressful situations, as well as for predator avoidance [24,65]. Thus, social interaction and shoaling tests were conducted to assess the differences between each zebrafish strain’s sociability by observing intraspecific interactions and their capability to form a shoal. After the data analysis, the PET zebrafish was found to less frequently interact with their conspecific shoal and formed a looser shoal than their AB strain counterpart. This phenomenon is reasonable, considering that this zebrafish resembles the wild zebrafish more than the domesticated one, and wild zebrafish are more shy and distressed in captivity compared to domesticated zebrafish strains, which was the AB strain in this case [66]. In addition, zebrafish sometimes form much larger groups in the wild and their shoaling behavior varies greatly across populations [67]. This result also complements another prior study by Robison et al., which demonstrated the existence of behavioral differences between wild and laboratory populations [47]. Besides, a study in a series of recently caught from the wild mouse strains that exhibited much greater polymorphism than standard laboratory strains also diverged in assorted behavioral assays [68]. Taken together, this finding confirms that the WT lines are unique to each colony and potentially varied in the degree of genetic diversity, since zebrafish are not maintained by a field-wide standard [69]. Then, less interaction with the conspecific was also displayed by the WIK strain. This finding is quite similar to previous studies that found the absence of their preference in a social novelty preference test [22,70]. Together with the current result, these findings emphasize that discriminatory preferences for a novel or familiar fish vary between strains. Moreover, the less conspecific interaction exhibited by this fish might be due to high baseline anxiety possessed by WIK zebrafish as it was described before in another study, since increased anxiety affects zebrafish responses to the presence of the con-and hetero-specifics [71]. Moreover, from another prior study, a less sociality was also shown by this fish during the shoaling test, another social-related test, which is consistent with the present study. In their study, this phenomenon was displayed by the less sociality in the shoal preference test [22,70]. The explanation for this finding is speculative at this point but demonstrates how the high inter-strain genetic polymorphisms of this strain are likely to exert different behaviors [72,73]. Furthermore, even though the PET zebrafish might also possess a genetic polymorphism, these two groups exhibited different behaviors compared to each other. These differences might have occurred since the polymorphisms that were discovered between and within species may be differentiated by alleles, which later, can affect the functional effects on neural transmission and influence their behaviors [74]. However, it is interesting to conduct a follow-up study in the future to determine the differences between the current PET zebrafish behavior and their behavior after a few generations in the current zebrafish facility. Since by that time, they may already become domesticated animals, we hypothesized that these generations will behave more similarly to the AB and TL strain zebrafishes even though there are still significant differences that will be observed. This hypothesis is based on genetics and epigenetics, which are two of the important factors affecting the behavior of animals [75,76]. If the hypothesis is true, it will highlight the genomic imprinting from a standardized laboratory environment.

From the best of our knowledge, this is the first study to observe the absolute mutant strain behavior in the novel tank, mirror-biting, predator avoidance, conspecific social interaction, and shoaling tests. Based on the results, this strain showed significantly different behaviors from the AB strain in almost all of the behavior tests, including the novel tank, mirror-biting, predator avoidance, and conspecific social interaction tests. Even though it is still a speculation, these differences might be related to the lack of melanophores and iridophores possessed by this fish, causing an alteration in their visual responses as mentioned by Ren and colleagues. In their study, under dim or bright conditions or under low contrast stimulation, both *albino* and *ruby* mutants showed reduced visual responses [77]. Other evidence in the zebrafish study showed that zebrafish mutants defective in melanophore functions caused altered behaviors, particularly behavioral blindness [78]. Zebrafish mutants that lack melanophores also showed defects in the motor apparatus, brain degeneration, retinal degeneration, and some zebrafish even showed specific defects along the visual pathway [78]. This experiment is in line with the absolute zebrafish tested in our study that exhibited different behavior compared to the AB zebrafish, especially in their visual responses (Figure 2, Figure 3 and Figure 4). Another study also found that melanophores play a role in the visual development of zebrafish. Mutant zebrafish that had a defect in their melanophores also showed a retinal degeneration and ultimately caused a visual impairment [79,80,81]. In addition, another study found increased bottom-dwelling in albino strain zebrafish, in relation to wild-type, which is in agreement with the current result [21,82]. Another research in zebrafish albino mutants showed that zebrafish mutants are blind at larval stages and gradually develop better visuals, however, adults zebrafish still have impaired vision [78]. Moreover, the deficiency of melanophores is also found to be related to the visual background adaptation response (VBA), a neuroendocrine camouflage response that matches pigment distribution in the body to ambient light levels, which may be altered in this absolute mutant strain [83]. Moreover, another prior study discovered that pigment cell interactions, which define the color pattern of the adult zebrafish, require a specific receptor function in a specific brain region. Therefore, there is a possibility that a lack of pigment in absolute zebrafish is related to several receptors in their brain and may involve in their behavior differences from the AB strain, which possesses normal pigmentation [84]. Besides, supporting these findings, other studies also demonstrated that differences in pigment patterns may be associated with other morphological or behavioral differences between fish, including predation avoidance, species recognition, and mate choice [85,86].

Then, the coefficient of variation of each zebrafish strain from every behavioral test was calculated to observe their reproducibility. From Supplementary Table S4, we found that the average lowest coefficient of variation was possessed by the AB zebrafish group, highlighting the strong reproducibility of these behaviors in this fish. Remarkably, the TL zebrafish also showed a comparable average coefficient of variation compared to the AB zebrafish, which also indicated the strong reproducibility and the robustness of the behaviors tested in the TL zebrafish. A high level of coefficient of variation can be a real problem to obtain significant results. As the impact of this condition, the high sample size would have to be used, which means that more animals would be used and this might not be trivial when getting animals from the wild besides being an ethical issue [87]. Furthermore, based on this table, a specific endpoint for each behavior test that exhibited the lowest and highest coefficient of variation can be revealed. In the novel tank test, the swimming time movement ratio possessed the lowest coefficient of variation in all of the zebrafish strains, while the highest one was varied. Meanwhile, in the other tests, except for the social interaction test, each strain had the same pattern regarding the levels of coefficient of variation. In the mirror-biting test, the longest duration in the mirror percentage had a higher level of coefficient of variation than another endpoint while in the predator avoidance test, the approaching predator time percentage was the endpoint that possessed the highest level of coefficient of variation. Furthermore, the average shoal area became the endpoint with the highest coefficient of variation, leaving the average furthest neighbor distance with the lowest one in the shoaling test. On the other hand, in the social interaction test, even though every fish showed the highest coefficient of variation in the longest conspecific interaction percentage, only absolute and WIK zebrafish displayed an average distance to the conspecific’s separator as the endpoint with the lowest coefficient of variation. Then, to find if the *n* number can affect the coefficient of variation of each zebrafish strain, a comparison was made as shown in Supplementary Figure S2, with three variations of the *n* number, which are 10, 20, and 30. From the figure, a persistent average coefficient of variation was displayed by the AB zebrafish (black) when 10, 20, and 30 *n* numbers were used, ranging from 65 to 70%. In contrast with AB zebrafish, the absolute, golden, PET, and the WIK zebrafishes showed an elevated average coefficient of variation, together with the increment of the *n* number. Interestingly, the TL zebrafish displayed a different pattern of the average coefficient of variation in relation to the *n* number. Taken together, this finding supports the strong reproducibility of the AB zebrafish, even when a not too high *n* number is used in the current behavioral tests, without neglecting the importance of a high *n* number in the behavior test. Thus, in the future zebrafish-based behavioral studies, we strongly suggest that the AB strain can be used as a standard strain, due to its more robust behavioral performance and less intra-strain individual variation than the rest of the strain tested in this experiment. However, it does not spontaneously omit the importance and usefulness of other fish strains, such as absolute zebrafish as a high-anxiety fish that is different from the AB zebrafish. Nevertheless, one has to keep in mind about the *n* number determination before conducting the experiment with those fish strains. In addition, since the WIK/AB crossed progeny is often to be used for genetic mapping crosses, it is interesting to carry out a future study in the behavior of the progeny to find out whether they possessed a different level of coefficient of variation to their ancestors and more similar behavior to the AB strain zebrafish. The result of this future experiment may help us to elucidate the potential for genetic variation and epigenetics in behavior studies.

Overall, each strain tested in this experiment showed specifically different behaviors with the AB zebrafish as summarized in Table 1. Furthermore, confirming this result, the PCA and heatmap clustering displayed two major clusters, showing the correlations and the distance between each strain regarding the behavioral test results. However, the exact mechanisms that underlie the differences we observed between these zebrafish strains remain unknown. Future works can be conducted by measuring the relative content of neurotransmitter and enzyme activities in the zebrafish brain to provide molecular mechanisms at either the mRNA or protein levels. Furthermore, there are some slight differences regarding the results found in the current study with other prior studies, including the differences regarding the comparison of AB and TL zebrafish behaviors. In their study, the cohesion and exploratory behavior of the TL zebrafish group were always stronger and higher, respectively, than those of the groups of AB zebrafish while in our study, both of them showed a similar shoal size and exploratory behavior [19,88]. Furthermore, from the previous study, WIK zebrafish showed a lower time in the upper half of the tank in the novel tank test which indicates anxiety, and this phenomenon was not displayed in the current experiment [89]. These conflicting results are probably due to the differences in methodology [48]. In addition, the group size and tank size may also have an effect on these differences [59]. This variation also highlights the importance to standardize experimental protocol, instrument setting, and software to perform zebrafish behaviors in the coming future. Furthermore, one has to remember that in the present study, mixed gender and different ages, ranging from 6 to 12 months old, of zebrafish were used. Since different gender and age ranges in zebrafish may cause the differences in their behavior, further studies are needed to be conducted in the future to investigate the effect of those two factors in zebrafish from different strains [90].

To sum up, the current findings emphasize the importance of using an appropriate zebrafish strain for behavioral studies, since zebrafish behavioral tests are being increasingly used in diverse disciplines. In addition, these findings also add to the growing database of phenotypical differences between the zebrafish of the several common strains used.

## Figures and Tables

**Table 1:** The summary of several zebrafish strains’ behavioral differences compared with the AB zebrafish. The signatures of the zebrafish behavioral tests are summarized (↑: upregulated, ↓: down-regulated, numbers of the arrow represent the number of altered behavior endpoints from each behavioral test). TL = Tübingen Long Fin, PET = Pet Store-purchased, WIK = Wild Indian Karyotype.

	Multiple Fish Behavior Tests					
Fish Strains	Locomotor Activity	Exploratory Behavior	Aggressiveness	Predator Avoidance	Conspecific Social Interaction	Shoaling Size
Absolute Zebrafish	-	↓↓↓↓	↓↓	↓↓	↓↓↓	-
TL Zebrafish	-	-	↓	↓	-	-
Golden Zebrafish	↑↑	↓↓↓	↑	-	-	-
PET Zebrafish	↑↑	↓↓↓↓↓	-	-	↓↓	↑↑
WIK Zebrafish	-	↓	↓	↓↓	↓↓↓	↑
*Puntigrus tetrazona*	↑↑	↑↑↑	↑↑	↓↓	↓↓	↓

## Supplementary Materials

The following are available online at https://www.mdpi.com/2079-7737/9/8/200/s1, Figure S1. Locomotor activity endpoint comparisons between the AB zebrafish (white), absolute zebrafish (red), TL zebrafish (blue), golden zebrafish (green), PET zebrafish (purple), and the WIK zebrafish (orange) in the predator avoidance test. Figure S2. Comparison of the average coefficient of variation of the six different zebrafish strains (AB (black), absolute (red), TL (blue), golden (green), PET (purple), WIK (orange)) in the six zebrafish behavior tests with three different *n* numbers. Table S1. Summary of the fish behavioral endpoints measured in each behavioral test during this experiment. Table S2. The mean, median, and standard deviation (SD) of the behavior endpoints of each fish group in every time interval from the novel tank test result. Table S3. The mean, median, and standard deviation (SD) of the *Puntigrus tetrazona* behavior endpoints in each behavioral test. Table S4. Summary of each fish strain coefficient of variation (CV) and standard deviation (SD) in each behavior endpoints.
