# Supplementary material for: Which Zebrafish Strains Are More Suitable to Perform Behavioral Studies? A Comprehensive Comparison by Phenomic Approach
Source: Biology (Basel). 2020 Aug 1;9(8):200. doi: 10.3390/biology9080200 (PMC7465594; doi:10.3390/biology9080200)
Supplement: Supplementary file 1 [file biology-09-00200-s001.zip › Table S4.docx]

**Table S4.** Summary of each fish strain coefficient of variation (CV) and standard deviation (SD) in each behavior endpoints.

| **Index** | **AB**  **Zebrafish** | | **Absolute Zebrafish** | | **TL**  **Zebrafish** | | **Golden Zebrafish** | | **PET Zebrafish** | | **WIK Zebrafish** | |
| --- | --- | --- | --- | --- | --- | --- | --- | --- | --- | --- | --- | --- |
|  | **CV (%)** | **SD** | **CV (%)** | **SD** | **CV (%)** | **SD** | **CV (%)** | **SD** | **CV (%)** | **SD** | **CV (%)** | **SD** |
| 1-1-1 | 30.18 | 1.23 | 38.07 | 1.67 | 28.80 | 1.30 | 45.33 | 2.21 | 37.66 | 1.93 | 51.75 | 1.90 |
| 1-1-2 | 85.28 | 9.94 | 138.73 | 18.35 | 99.46 | 8.96 | 138.33 | 24.36 | 126.17 | 18.22 | 96.15 | 22.95 |
| 1-1-3 | 11.72 | 9.76 | 23.26 | 18.54 | 11.21 | 9.56 | 31.95 | 21.77 | 25.04 | 17.06 | 41.04 | 22.31 |
| 1-1-4 | 119.26 | 4.65 | 124.95 | 6.73 | 98.82 | 5.62 | 95.71 | 11.26 | 83.11 | 12.22 | 99.99 | 7.37 |
| 1-1-5 | 94.04 | 26.07 | 227.21 | 10.59 | 86.04 | 23.20 | 152.74 | 21.47 | 239.74 | 8.41 | 148.35 | 16.88 |
| 1-1-6 | 82.26 | 2.82 | 217.53 | 2.59 | 76.28 | 3.82 | 126.22 | 3.27 | 202.30 | 2.56 | 128.98 | 3.93 |
| 1-1-7 | 150.18 | 17.83 | 50.79 | 19.38 | 135.72 | 20.95 | 94.60 | 23.48 | 68.98 | 21.14 | 104.50 | 22.50 |
| 1-1-8 | 99.42 | 73.85 | 242.31 | 45.28 | 84.76 | 66.83 | 177.96 | 85.09 | 218.79 | 33.10 | 151.91 | 61.23 |
| 1-1-9 | 33.20 | 1.49 | 26.80 | 1.15 | 26.67 | 1.33 | 32.85 | 1.80 | 24.26 | 1.34 | 67.84 | 3.91 |
| 1-2-1 | 36.20 | 1.44 | 39.72 | 1.69 | 38.34 | 1.47 | 33.47 | 1.77 | 41.25 | 1.82 | 35.29 | 1.70 |
| 1-2-2 | 71.59 | 7.53 | 126.70 | 18.16 | 104.65 | 14.07 | 126.99 | 13.92 | 110.43 | 16.10 | 106.77 | 13.18 |
| 1-2-3 | 8.83 | 7.48 | 24.15 | 19.27 | 16.97 | 13.96 | 19.88 | 15.60 | 21.00 | 16.31 | 15.88 | 12.58 |
| 1-2-4 | 153.56 | 4.74 | 128.68 | 6.02 | 166.17 | 4.12 | 95.51 | 10.35 | 117.36 | 7.48 | 94.76 | 7.78 |
| 1-2-5 | 61.45 | 25.26 | 125.10 | 20.29 | 80.25 | 27.05 | 81.69 | 25.36 | 154.83 | 15.24 | 66.38 | 24.94 |
| 1-2-6 | 78.05 | 3.84 | 111.86 | 3.88 | 76.60 | 2.67 | 87.24 | 5.20 | 144.70 | 3.28 | 76.39 | 5.87 |
| 1-2-7 | 164.92 | 13.26 | 95.63 | 25.55 | 140.44 | 23.92 | 146.82 | 23.01 | 77.50 | 25.48 | 214.28 | 17.06 |
| 1-2-8 | 70.03 | 76.82 | 140.85 | 84.39 | 78.22 | 63.11 | 90.17 | 102.86 | 151.18 | 50.47 | 69.14 | 85.36 |
| 1-2-9 | 35.58 | 1.80 | 32.08 | 1.48 | 23.07 | 1.18 | 32.51 | 2.07 | 24.25 | 1.35 | 31.00 | 2.03 |
| 2-1 | 47.42 | 18.06 | 91.46 | 17.81 | 53.70 | 22.54 | 40.95 | 22.98 | 66.05 | 28.24 | 65.99 | 23.02 |
| 2-2 | 83.05 | 9.12 | 91.96 | 1.79 | 91.23 | 5.62 | 128.60 | 18.92 | 124.60 | 12.26 | 80.51 | 4.27 |
| 3-1 | 137.00 | 3.46 | 141.50 | 9.17 | 152.60 | 6.38 | 128.10 | 9.59 | 179.30 | 12.93 | 63.82 | 26.48 |
| 3-2 | 13.84 | 1.40 | 14.87 | 1.29 | 14.09 | 1.24 | 22.30 | 2.19 | 23.20 | 2.32 | 40.23 | 2.47 |
| 4-1 | 33.86 | 24.84 | 51.60 | 22.36 | 24.88 | 19.84 | 38.24 | 27.84 | 51.95 | 28.16 | 44.77 | 23.26 |
| 4-2 | 55.24 | 1.47 | 27.93 | 1.09 | 53.21 | 1.17 | 62.35 | 1.63 | 55.49 | 2.32 | 36.97 | 1.34 |
| 4-3 | 96.13 | 22.88 | 123.60 | 9.64 | 83.75 | 14.09 | 88.73 | 17.91 | 118.50 | 19.73 | 100.90 | 9.45 |
| 5-1 | 20.73 | 0.95 | 29.90 | 1.47 | 26.46 | 1.19 | 32.12 | 1.45 | 25.12 | 1.35 | 26.28 | 1.37 |
| 5-2 | 46.02 | 1.92 | 62.59 | 2.64 | 38.72 | 1.38 | 50.03 | 1.87 | 55.86 | 1.58 | 58.17 | 2.73 |
| 5-3 | 20.73 | 0.66 | 29.90 | 1.00 | 27.29 | 0.84 | 31.90 | 0.98 | 27.13 | 0.93 | 31.23 | 1.09 |
| 5-4 | 19.33 | 1.16 | 25.67 | 1.66 | 21.60 | 1.28 | 29.59 | 1.77 | 18.23 | 1.33 | 17.92 | 1.24 |
| **Average** | **67.56** | **12.96** | **89.84** | **12.93** | **67.59** | **12.71** | **78.03** | **17.31** | **90.14** | **12.57** | **74.73** | **14.83** |
