# Supplementary material for: Which Zebrafish Strains Are More Suitable to Perform Behavioral Studies? A Comprehensive Comparison by Phenomic Approach
Source: Biology (Basel). 2020 Aug 1;9(8):200. doi: 10.3390/biology9080200 (PMC7465594; doi:10.3390/biology9080200)
Supplement: Supplementary file 1 [file biology-09-00200-s001.zip › Figure S1.docx]

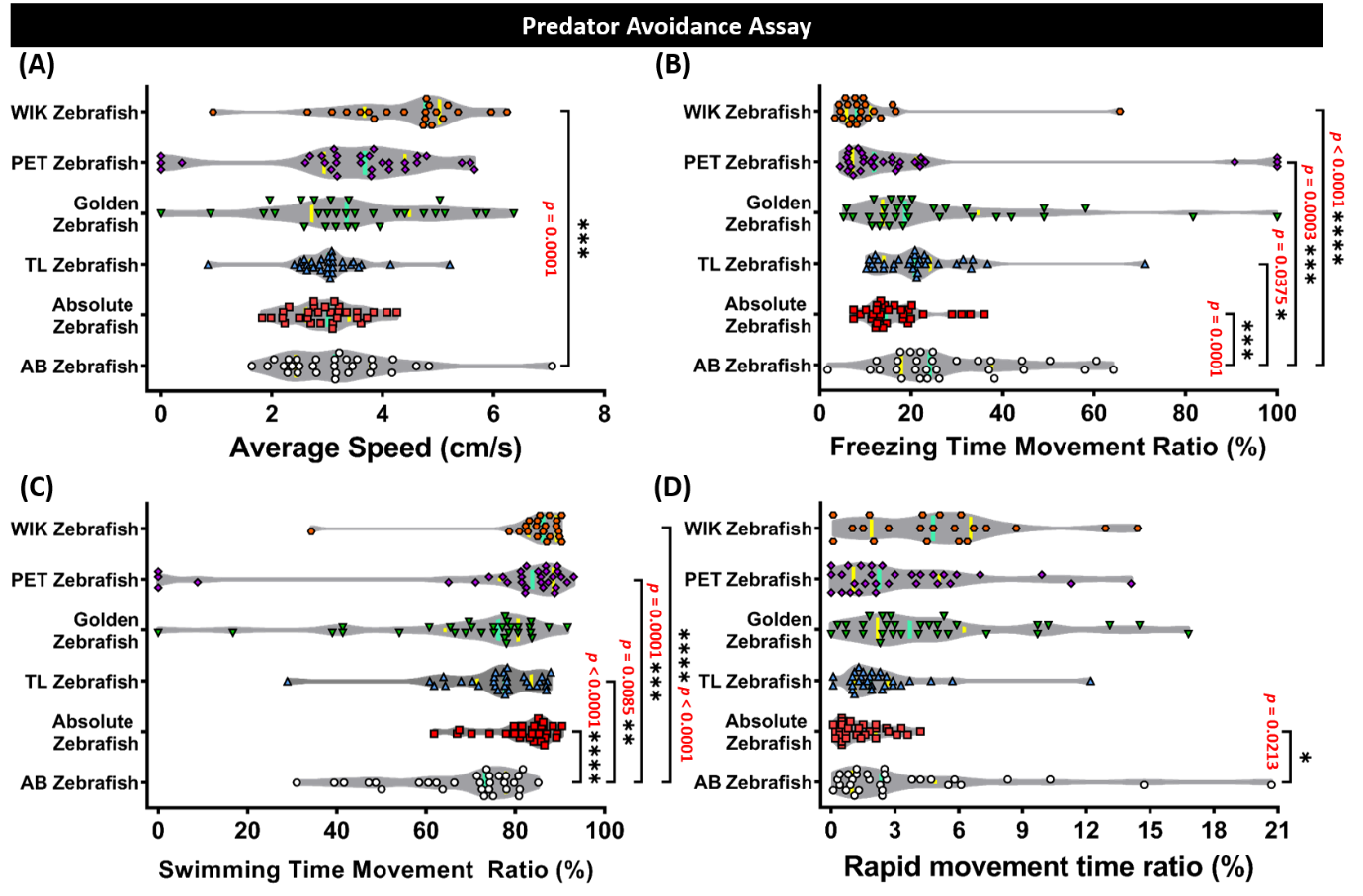


**Figure S1.** Locomotor activity endpoints comparisons between AB zebrafish (white), absolute zebrafish (red), TL zebrafish (blue), golden zebrafish (green), PET zebrafish (purple), and WIK zebrafish (orange) in the predator avoidance test. (A) Average speed, (B) freezing time movement ratio, (C) swimming time movement ratio, and (D) rapid movement time ratio were analyzed for locomotor activity assay. The median and the interquartile for the violin plot were labeled with the bold line colored with cyan and yellow, respectively. The data were analyzed by a Mann-Whitney test (n = 30 for each group, except for WIK zebrafish (n = 21); * p < 0.05; ** p < 0.01;*** p < 0.001; **** *P*< 0.0001).
