# Supplementary material for: Which Zebrafish Strains Are More Suitable to Perform Behavioral Studies? A Comprehensive Comparison by Phenomic Approach
Source: Biology (Basel). 2020 Aug 1;9(8):200. doi: 10.3390/biology9080200 (PMC7465594; doi:10.3390/biology9080200)
Supplement: Supplementary file 1 [file biology-09-00200-s001.zip › Figure S2.docx]

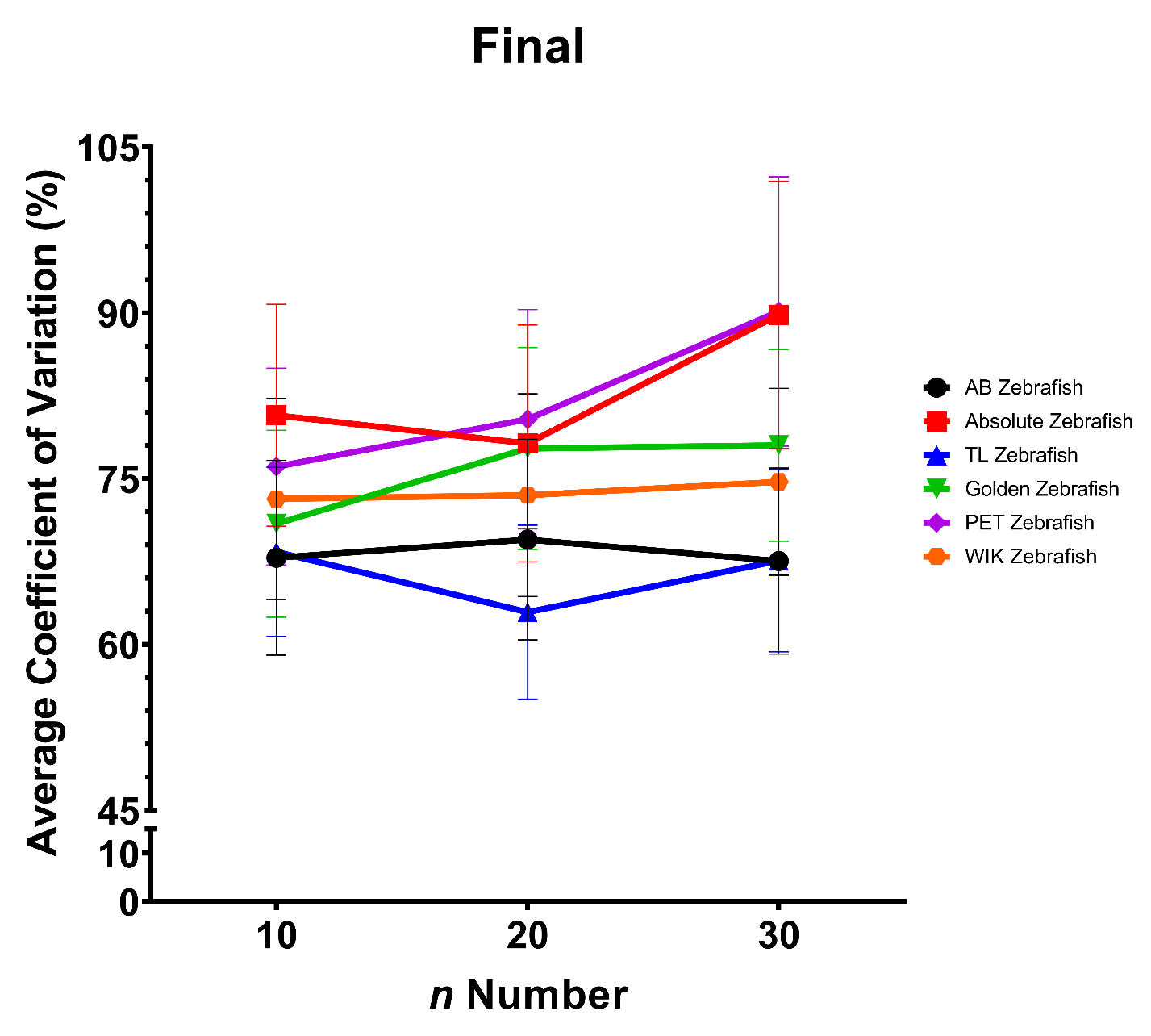


**Figure S2.** Comparison of the average coefficient of variation of six different zebrafish strains (AB (black), absolute (red), TL (blue), golden (green), PET (purple), WIK (orange)) in six zebrafish behavior tests with three different *n* numbers.
