# Supplementary material for: Which Zebrafish Strains Are More Suitable to Perform Behavioral Studies? A Comprehensive Comparison by Phenomic Approach
Source: Biology (Basel). 2020 Aug 1;9(8):200. doi: 10.3390/biology9080200 (PMC7465594; doi:10.3390/biology9080200)
Supplement: Supplementary file 1 [file biology-09-00200-s001.zip › Table S1.docx]

**Table S1.** Summary of fish behavioral endpoints measured in each behavioral test during this experiment.

| **Index** | **Behavior endpoints (units)** | **Definition** | **Applied to** |
| --- | --- | --- | --- |
| **1-1-1 & 1-2-1** | Average speed (cm s^-1^) | Total distance traveled by fish divided by total time duration | **Novel Tank Test** |
| **1-1-2 & 1-2-2** | Freezing time movement ratio (%) | Total percentage of time when fish’s speed less than 1 cm s^-1^ |  |
| **1-1-3 & 1-2-3** | Swimming time movement ratio (%) | Total percentage of time when fish’s speed is between 1 and 10 cm s^-1^ |  |
| **1-1-4 & 1-2-4** | Rapid movement ratio (%) | Total percentage of time when fish’s speed more than 10 cm s^-1^ |  |
| **1-1-5 & 1-2-5** | Time in top duration (%) | Total time spent in the top portion of the novel tank in percentage |  |
| **1-1-6 & 1-2-6** | Number of entries to the top | Total times fish enter the upper half of the tank |  |
| **1-1-7 & 1-2-7** | Latency to enter the top (s) | The amount of time it takes the fish to cross into the upper half of the tank |  |
| **1-1-8 & 1-2-8** | Total distance traveled in the top (cm) | Total distance traveled in the top portion of the novel tank |  |
| **1-1-9 & 1-2-9** | Thigmotaxis (cm) | The average distance of the fish from the center of the tank |  |
| **2-1** | Mirror biting time percentage (%) | Total percentage of time when fish stayed in the mirror biting zone | **Mirror Biting Test** |
| **2-2** | Longest duration in the mirror side percentage (%) | Total percentage of fish longest duration stayed in the mirror biting zone |  |
| **3-1** | Approaching predator time percentage (%) | Total percentage of time when fish stayed in the approaching predator zone | **Predator Avoidance Test** |
| **3-2** | Average distance to the predator’s separator (cm) | Average distance of fish to the predator’s separator |  |
| **4-1** | Conspecific interaction time percentage (%) | Total percentage of time when fish stayed in the conspecific interaction zone | **Social Interaction Test** |
| **4-2** | Average distance to the conspecific’s separator (cm) | Average distance of fish to the conspecific’s separator |  |
| **4-3** | Longest conspecific interaction percentage (%) | Total percentage of fish longest duration stayed in the conspecific interaction |  |
| **5-1** | Average inter-fish distance (cm) | Average distance between the body center of every member of the shoal | **Shoaling Test** |
| **5-2** | Average shoal area (cm^2^) | Average size of the shoal |  |
| **5-3** | Average nearest neighbor distance (cm) | Distance for the body center of each fish to the closest neighboring fish |  |
| **5-4** | Average farthest neighbor distance (cm) | Distance for the body center of each fish to the farthest neighboring fish |  |
