# Supplementary material for: Which Zebrafish Strains Are More Suitable to Perform Behavioral Studies? A Comprehensive Comparison by Phenomic Approach
Source: Biology (Basel). 2020 Aug 1;9(8):200. doi: 10.3390/biology9080200 (PMC7465594; doi:10.3390/biology9080200)
Supplement: Supplementary file 1 [file biology-09-00200-s001.zip › Table S2.docx]

**Table S2.** The mean, median, and SD of behavior endpoints of each fish group in every time interval from the novel tank test result.

| Zebrafish Line | Novel Tank Test Endpoint | Time Interval (min) | Mean | Median | SD |
| --- | --- | --- | --- | --- | --- |
| **AB Zebrafish** | Average Speed (cm/s) | 0 - 1 | 4.04 | 3.76 | 1.24 |
|  |  | 5 - 6 | 4.12 | 3.94 | 1.32 |
|  |  | 10 - 11 | 4.01 | 3.63 | 1.12 |
|  |  | 15 - 16 | 3.93 | 3.73 | 1.22 |
|  |  | 20 - 21 | 4.04 | 4.07 | 1.52 |
|  |  | 25 – 26 | 4.03 | 3.61 | 1.50 |
|  |  | 30 - 31 | 3.92 | 3.53 | 1.52 |
|  | Freezing Time Movement Ratio (%) | 0 - 1 | 13.60 | 8.40 | 17.39 |
|  |  | 5 - 6 | 9.82 | 7.55 | 7.76 |
|  |  | 10 - 11 | 9.54 | 8.90 | 4.67 |
|  |  | 15 - 16 | 9.85 | 8.55 | 5.49 |
|  |  | 20 - 21 | 10.63 | 7.15 | 9.39 |
|  |  | 25 – 26 | 10.49 | 8.10 | 7.92 |
|  |  | 30 - 31 | 10.95 | 9.50 | 7.31 |
|  | Swimming Time Movement Ratio (%) | 0 - 1 | 81.70 | 87.55 | 16.77 |
|  |  | 5 - 6 | 85.22 | 87.35 | 7.69 |
|  |  | 10 - 11 | 86.05 | 87.40 | 4.83 |
|  |  | 15 - 16 | 85.92 | 86.65 | 5.10 |
|  |  | 20 - 21 | 84.38 | 86.65 | 9.49 |
|  |  | 25 – 26 | 84.51 | 86.20 | 7.91 |
|  |  | 30 - 31 | 84.18 | 86.00 | 7.40 |
|  | Rapid Time Movement Ratio (%) | 0 - 1 | 4.67 | 3.70 | 4.69 |
|  |  | 5 - 6 | 4.93 | 2.40 | 6.45 |
|  |  | 10 - 11 | 4.36 | 2.90 | 5.52 |
|  |  | 15 - 16 | 4.20 | 2.35 | 5.77 |
|  |  | 20 - 21 | 4.95 | 2.60 | 8.01 |
|  |  | 25 – 26 | 4.97 | 2.30 | 7.59 |
|  |  | 30 - 31 | 4.84 | 2.20 | 7.85 |
|  | Time in Top Duration (%) | 0 - 1 | 20.55 | 1.82 | 34.29 |
|  |  | 5 - 6 | 37.05 | 32.80 | 23.67 |
|  |  | 10 - 11 | 39.46 | 43.62 | 20.26 |
|  |  | 15 - 16 | 41.10 | 40.68 | 24.28 |
|  |  | 20 - 21 | 38.36 | 34.39 | 23.63 |
|  |  | 25 – 26 | 46.81 | 46.90 | 28.02 |
|  |  | 30 - 31 | 38.46 | 36.97 | 25.09 |
|  | Number of Entries to The Top | 0 - 1 | 2.07 | 1.00 | 2.74 |
|  |  | 5 - 6 | 4.60 | 4.00 | 2.98 |
|  |  | 10 - 11 | 5.57 | 5.00 | 2.75 |
|  |  | 15 - 16 | 4.17 | 3.00 | 2.59 |
|  |  | 20 - 21 | 4.37 | 3.50 | 3.49 |
|  |  | 25 – 26 | 5.37 | 4.50 | 4.94 |
|  |  | 30 - 31 | 5.57 | 4.00 | 4.35 |
|  | Latency to Enter the Top (s) | 0 - 1 | 36.24 | 54.65 | 27.41 |
|  |  | 5 - 6 | 8.17 | 2.07 | 15.25 |
|  |  | 10 - 11 | 5.76 | 0.65 | 10.84 |
|  |  | 15 - 16 | 10.92 | 3.20 | 15.49 |
|  |  | 20 - 21 | 7.71 | 1.96 | 14.50 |
|  |  | 25 – 26 | 8.24 | 0.16 | 15.47 |
|  |  | 30 - 31 | 5.34 | 0.49 | 7.59 |
|  | Total Distance Traveled In The Top (cm) | 0 - 1 | 49.82 | 7.53 | 81.65 |
|  |  | 5 - 6 | 102.37 | 80.84 | 80.87 |
|  |  | 10 - 11 | 106.61 | 110.03 | 59.03 |
|  |  | 15 - 16 | 105.20 | 93.30 | 68.24 |
|  |  | 20 - 21 | 102.54 | 91.64 | 69.54 |
|  |  | 25 – 26 | 129.02 | 97.83 | 99.82 |
|  |  | 30 - 31 | 99.48 | 89.43 | 69.67 |
|  | Thigmotaxis (cm) | 0 - 1 | 3.68 | 3.33 | 1.25 |
|  |  | 5 - 6 | 4.46 | 4.15 | 1.26 |
|  |  | 10 - 11 | 5.22 | 4.79 | 1.95 |
|  |  | 15 - 16 | 4.80 | 4.63 | 1.51 |
|  |  | 20 - 21 | 4.97 | 4.37 | 1.69 |
|  |  | 25 – 26 | 5.19 | 4.67 | 1.95 |
|  |  | 30 - 31 | 5.20 | 4.24 | 2.05 |
| **Absolute Zebrafish** | Average Speed (cm/s) | 0 - 1 | 4.16 | 3.93 | 1.64 |
|  |  | 5 - 6 | 4.30 | 4.02 | 1.50 |
|  |  | 10 - 11 | 4.68 | 4.61 | 1.87 |
|  |  | 15 - 16 | 4.85 | 4.51 | 1.76 |
|  |  | 20 - 21 | 4.19 | 4.22 | 1.60 |
|  |  | 25 – 26 | 3.83 | 4.19 | 1.58 |
|  |  | 30 - 31 | 4.22 | 4.25 | 1.82 |
|  | Freezing Time Movement Ratio (%) | 0 - 1 | 16.20 | 9.90 | 20.82 |
|  |  | 5 - 6 | 12.95 | 7.30 | 16.95 |
|  |  | 10 - 11 | 11.02 | 7.10 | 17.27 |
|  |  | 15 - 16 | 9.36 | 6.65 | 8.98 |
|  |  | 20 - 21 | 14.08 | 7.25 | 17.42 |
|  |  | 25 – 26 | 16.95 | 6.50 | 24.26 |
|  |  | 30 - 31 | 15.28 | 7.20 | 21.98 |
|  | Swimming Time Movement Ratio (%) | 0 - 1 | 77.33 | 85.40 | 20.32 |
|  |  | 5 - 6 | 81.07 | 86.75 | 17.02 |
|  |  | 10 - 11 | 81.16 | 87.55 | 18.28 |
|  |  | 15 - 16 | 82.11 | 87.95 | 15.07 |
|  |  | 20 - 21 | 80.75 | 87.45 | 16.90 |
|  |  | 25 – 26 | 79.00 | 86.25 | 22.78 |
|  |  | 30 - 31 | 78.31 | 86.90 | 22.32 |
|  | Rapid Time Movement Ratio (%) | 0 - 1 | 6.45 | 4.15 | 7.50 |
|  |  | 5 - 6 | 5.95 | 3.10 | 7.04 |
|  |  | 10 - 11 | 7.79 | 4.30 | 10.93 |
|  |  | 15 - 16 | 8.52 | 3.70 | 11.33 |
|  |  | 20 - 21 | 5.16 | 2.80 | 7.03 |
|  |  | 25 – 26 | 4.01 | 2.65 | 4.40 |
|  |  | 30 - 31 | 6.39 | 3.35 | 8.67 |
|  | Time in Top Duration (%) | 0 - 1 | 0.37 | 0.00 | 1.41 |
|  |  | 5 - 6 | 8.09 | 1.82 | 12.80 |
|  |  | 10 - 11 | 12.03 | 2.43 | 17.57 |
|  |  | 15 - 16 | 19.78 | 13.07 | 21.63 |
|  |  | 20 - 21 | 13.30 | 4.10 | 19.63 |
|  |  | 25 – 26 | 14.61 | 8.04 | 17.62 |
|  |  | 30 - 31 | 18.12 | 7.14 | 22.27 |
|  | Number of Entries to The Top | 0 - 1 | 0.30 | 0.00 | 1.15 |
|  |  | 5 - 6 | 2.00 | 1.00 | 2.73 |
|  |  | 10 - 11 | 2.93 | 2.00 | 3.90 |
|  |  | 15 - 16 | 4.30 | 3.00 | 4.15 |
|  |  | 20 - 21 | 3.13 | 2.00 | 4.19 |
|  |  | 25 – 26 | 3.00 | 3.00 | 3.12 |
|  |  | 30 - 31 | 3.60 | 2.00 | 4.07 |
|  | Latency to Enter the Top (s) | 0 - 1 | 57.56 | 60.00 | 8.79 |
|  |  | 5 - 6 | 37.76 | 42.62 | 24.20 |
|  |  | 10 - 11 | 34.43 | 40.96 | 25.13 |
|  |  | 15 - 16 | 22.49 | 11.68 | 24.79 |
|  |  | 20 - 21 | 34.90 | 37.84 | 24.17 |
|  |  | 25 – 26 | 30.57 | 20.74 | 27.30 |
|  |  | 30 - 31 | 22.80 | 7.84 | 25.93 |
|  | Total Distance Traveled In The Top (cm) | 0 - 1 | 1.32 | 0.00 | 5.17 |
|  |  | 5 - 6 | 28.94 | 6.57 | 49.28 |
|  |  | 10 - 11 | 49.58 | 7.64 | 81.39 |
|  |  | 15 - 16 | 77.18 | 45.71 | 114.75 |
|  |  | 20 - 21 | 45.22 | 10.09 | 73.10 |
|  |  | 25 – 26 | 50.23 | 31.67 | 58.93 |
|  |  | 30 - 31 | 66.87 | 19.59 | 90.78 |
|  | Thigmotaxis (cm) | 0 - 1 | 4.21 | 3.85 | 1.24 |
|  |  | 5 - 6 | 4.23 | 4.20 | 1.03 |
|  |  | 10 - 11 | 4.48 | 4.44 | 1.19 |
|  |  | 15 - 16 | 4.72 | 4.75 | 1.23 |
|  |  | 20 - 21 | 4.43 | 4.32 | 1.43 |
|  |  | 25 – 26 | 4.76 | 3.97 | 1.89 |
|  |  | 30 - 31 | 4.56 | 4.31 | 1.38 |
| **TL Zebrafish** | Average Speed (cm/s) | 0 - 1 | 4.64 | 4.74 | 1.31 |
|  |  | 5 - 6 | 4.56 | 4.48 | 1.21 |
|  |  | 10 - 11 | 4.40 | 4.29 | 1.39 |
|  |  | 15 - 16 | 4.20 | 4.00 | 1.51 |
|  |  | 20 - 21 | 3.73 | 3.69 | 1.33 |
|  |  | 25 – 26 | 3.80 | 3.74 | 1.74 |
|  |  | 30 - 31 | 3.58 | 3.63 | 1.29 |
|  | Freezing Time Movement Ratio (%) | 0 - 1 | 8.37 | 6.85 | 7.53 |
|  |  | 5 - 6 | 9.80 | 6.20 | 11.33 |
|  |  | 10 - 11 | 8.65 | 6.45 | 8.03 |
|  |  | 15 - 16 | 12.19 | 7.35 | 13.70 |
|  |  | 20 - 21 | 13.36 | 11.30 | 12.71 |
|  |  | 25 – 26 | 14.68 | 9.90 | 17.29 |
|  |  | 30 - 31 | 13.47 | 9.15 | 12.56 |
|  | Swimming Time Movement Ratio (%) | 0 - 1 | 85.39 | 87.40 | 9.53 |
|  |  | 5 - 6 | 84.59 | 88.15 | 11.31 |
|  |  | 10 - 11 | 86.24 | 88.35 | 7.85 |
|  |  | 15 - 16 | 82.60 | 88.15 | 14.43 |
|  |  | 20 - 21 | 83.04 | 86.30 | 12.01 |
|  |  | 25 – 26 | 80.64 | 86.70 | 17.03 |
|  |  | 30 - 31 | 83.39 | 88.15 | 12.35 |
|  | Rapid Time Movement Ratio (%) | 0 - 1 | 6.21 | 4.75 | 5.87 |
|  |  | 5 - 6 | 5.58 | 4.70 | 4.64 |
|  |  | 10 - 11 | 5.08 | 2.75 | 6.04 |
|  |  | 15 - 16 | 5.17 | 2.65 | 7.06 |
|  |  | 20 - 21 | 3.56 | 2.10 | 5.14 |
|  |  | 25 – 26 | 4.65 | 1.70 | 8.78 |
|  |  | 30 - 31 | 3.10 | 1.75 | 6.03 |
|  | Time in Top Duration (%) | 0 - 1 | 19.15 | 13.69 | 20.36 |
|  |  | 5 - 6 | 31.33 | 31.58 | 26.04 |
|  |  | 10 - 11 | 33.77 | 32.70 | 23.19 |
|  |  | 15 - 16 | 37.39 | 38.97 | 27.54 |
|  |  | 20 - 21 | 35.91 | 35.20 | 27.95 |
|  |  | 25 – 26 | 31.17 | 25.42 | 27.75 |
|  |  | 30 - 31 | 31.02 | 34.79 | 24.96 |
|  | Number of Entries to The Top | 0 - 1 | 4.67 | 4.50 | 3.63 |
|  |  | 5 - 6 | 5.27 | 4.00 | 4.33 |
|  |  | 10 - 11 | 5.10 | 5.00 | 3.51 |
|  |  | 15 - 16 | 3.83 | 3.00 | 3.06 |
|  |  | 20 - 21 | 3.53 | 4.00 | 2.64 |
|  |  | 25 – 26 | 3.27 | 4.00 | 2.20 |
|  |  | 30 - 31 | 3.27 | 3.50 | 2.77 |
|  | Latency to Enter the Top (s) | 0 - 1 | 23.59 | 9.72 | 24.53 |
|  |  | 5 - 6 | 12.45 | 5.10 | 18.41 |
|  |  | 10 - 11 | 12.81 | 2.95 | 19.89 |
|  |  | 15 - 16 | 14.43 | 3.76 | 22.59 |
|  |  | 20 - 21 | 16.53 | 4.60 | 23.21 |
|  |  | 25 – 26 | 17.47 | 1.92 | 25.51 |
|  |  | 30 - 31 | 20.52 | 7.41 | 24.36 |
|  | Total Distance Traveled In The Top (cm) | 0 - 1 | 63.19 | 35.67 | 72.00 |
|  |  | 5 - 6 | 91.14 | 84.08 | 72.38 |
|  |  | 10 - 11 | 92.11 | 100.49 | 56.10 |
|  |  | 15 - 16 | 95.12 | 91.22 | 70.93 |
|  |  | 20 - 21 | 83.58 | 80.55 | 67.02 |
|  |  | 25 – 26 | 75.46 | 66.95 | 60.20 |
|  |  | 30 - 31 | 69.27 | 81.68 | 54.28 |
|  | Thigmotaxis (cm) | 0 - 1 | 4.13 | 4.07 | 1.24 |
|  |  | 5 - 6 | 5.62 | 5.81 | 1.33 |
|  |  | 10 - 11 | 5.40 | 5.23 | 1.43 |
|  |  | 15 - 16 | 5.25 | 5.44 | 1.34 |
|  |  | 20 - 21 | 4.89 | 5.17 | 1.16 |
|  |  | 25 – 26 | 5.38 | 5.54 | 1.19 |
|  |  | 30 - 31 | 4.94 | 5.05 | 1.04 |
| **Golden Zebrafish** | Average Speed (cm/s) | 0 - 1 | 4.18 | 3.99 | 2.64 |
|  |  | 5 - 6 | 5.42 | 5.70 | 2.01 |
|  |  | 10 - 11 | 5.55 | 5.83 | 1.99 |
|  |  | 15 - 16 | 5.49 | 5.44 | 1.30 |
|  |  | 20 - 21 | 5.23 | 5.07 | 1.83 |
|  |  | 25 – 26 | 5.25 | 5.06 | 1.70 |
|  |  | 30 - 31 | 5.27 | 4.91 | 2.26 |
|  | Freezing Time Movement Ratio (%) | 0 - 1 | 29.04 | 10.25 | 32.42 |
|  |  | 5 - 6 | 12.63 | 4.75 | 19.52 |
|  |  | 10 - 11 | 14.21 | 5.25 | 21.14 |
|  |  | 15 - 16 | 7.56 | 3.80 | 8.45 |
|  |  | 20 - 21 | 12.88 | 5.80 | 20.17 |
|  |  | 25 – 26 | 10.83 | 6.15 | 12.70 |
|  |  | 30 - 31 | 11.73 | 7.15 | 14.35 |
|  | Swimming Time Movement Ratio (%) | 0 - 1 | 62.18 | 74.95 | 29.47 |
|  |  | 5 - 6 | 75.39 | 81.60 | 17.52 |
|  |  | 10 - 11 | 72.69 | 78.10 | 18.32 |
|  |  | 15 - 16 | 81.39 | 83.55 | 9.63 |
|  |  | 20 - 21 | 76.51 | 83.80 | 19.35 |
|  |  | 25 – 26 | 79.06 | 85.60 | 15.79 |
|  |  | 30 - 31 | 78.51 | 84.35 | 17.63 |
|  | Rapid Time Movement Ratio (%) | 0 - 1 | 8.75 | 4.35 | 12.06 |
|  |  | 5 - 6 | 11.95 | 9.55 | 9.85 |
|  |  | 10 - 11 | 13.08 | 11.20 | 8.76 |
|  |  | 15 - 16 | 11.02 | 9.55 | 7.38 |
|  |  | 20 - 21 | 10.59 | 6.55 | 9.58 |
|  |  | 25 – 26 | 10.08 | 7.30 | 10.93 |
|  |  | 30 - 31 | 9.72 | 7.00 | 11.29 |
|  | Time in Top Duration (%) | 0 - 1 | 9.76 | 0.00 | 27.30 |
|  |  | 5 - 6 | 14.14 | 10.39 | 14.47 |
|  |  | 10 - 11 | 29.74 | 31.64 | 22.63 |
|  |  | 15 - 16 | 29.36 | 30.65 | 25.02 |
|  |  | 20 - 21 | 31.83 | 34.24 | 26.34 |
|  |  | 25 – 26 | 29.13 | 34.25 | 23.33 |
|  |  | 30 - 31 | 34.00 | 36.94 | 26.75 |
|  | Number of Entries to The Top | 0 - 1 | 0.70 | 0.00 | 1.58 |
|  |  | 5 - 6 | 4.50 | 5.00 | 3.56 |
|  |  | 10 - 11 | 6.33 | 7.00 | 4.69 |
|  |  | 15 - 16 | 5.30 | 6.00 | 4.28 |
|  |  | 20 - 21 | 6.47 | 6.00 | 5.34 |
|  |  | 25 – 26 | 6.27 | 5.50 | 5.81 |
|  |  | 30 - 31 | 5.77 | 5.00 | 5.35 |
|  | Latency to Enter the Top (s) | 0 - 1 | 48.92 | 60.00 | 21.75 |
|  |  | 5 - 6 | 24.38 | 15.19 | 24.11 |
|  |  | 10 - 11 | 17.50 | 4.95 | 24.59 |
|  |  | 15 - 16 | 20.19 | 3.99 | 25.99 |
|  |  | 20 - 21 | 10.61 | 2.20 | 18.59 |
|  |  | 25 – 26 | 15.82 | 1.13 | 23.25 |
|  |  | 30 - 31 | 17.75 | 5.87 | 24.20 |
|  | Total Distance Traveled In The Top (cm) | 0 - 1 | 30.32 | 0.00 | 110.56 |
|  |  | 5 - 6 | 60.08 | 60.17 | 57.95 |
|  |  | 10 - 11 | 119.30 | 123.99 | 86.77 |
|  |  | 15 - 16 | 107.88 | 97.02 | 98.95 |
|  |  | 20 - 21 | 115.69 | 114.32 | 93.46 |
|  |  | 25 – 26 | 102.61 | 90.55 | 89.38 |
|  |  | 30 - 31 | 128.27 | 108.60 | 129.64 |
|  | Thigmotaxis (cm) | 0 - 1 | 4.65 | 3.91 | 2.28 |
|  |  | 5 - 6 | 6.08 | 5.94 | 1.70 |
|  |  | 10 - 11 | 6.60 | 6.36 | 1.42 |
|  |  | 15 - 16 | 6.31 | 5.77 | 2.11 |
|  |  | 20 - 21 | 6.52 | 5.99 | 1.92 |
|  |  | 25 – 26 | 6.25 | 5.63 | 2.15 |
|  |  | 30 - 31 | 6.43 | 6.54 | 2.11 |
| **PET Zebrafish** | Average Speed (cm/s) | 0 - 1 | 5.05 | 5.75 | 2.80 |
|  |  | 5 - 6 | 5.51 | 5.53 | 1.39 |
|  |  | 10 - 11 | 4.94 | 4.93 | 1.60 |
|  |  | 15 - 16 | 4.60 | 4.40 | 1.93 |
|  |  | 20 - 21 | 4.72 | 4.24 | 1.64 |
|  |  | 25 – 26 | 4.08 | 4.27 | 1.50 |
|  |  | 30 - 31 | 4.31 | 3.40 | 2.21 |
|  | Freezing Time Movement Ratio (%) | 0 - 1 | 27.09 | 8.95 | 30.08 |
|  |  | 5 - 6 | 8.29 | 4.85 | 12.06 |
|  |  | 10 - 11 | 10.26 | 6.75 | 12.52 |
|  |  | 15 - 16 | 14.17 | 6.95 | 20.33 |
|  |  | 20 - 21 | 7.42 | 6.75 | 4.26 |
|  |  | 25 – 26 | 16.35 | 7.45 | 21.85 |
|  |  | 30 - 31 | 16.77 | 9.35 | 17.96 |
|  | Swimming Time Movement Ratio (%) | 0 - 1 | 57.73 | 59.35 | 23.08 |
|  |  | 5 - 6 | 79.81 | 83.10 | 13.23 |
|  |  | 10 - 11 | 80.06 | 85.50 | 14.85 |
|  |  | 15 - 16 | 77.32 | 85.70 | 19.89 |
|  |  | 20 - 21 | 84.87 | 86.35 | 7.35 |
|  |  | 25 – 26 | 78.08 | 85.05 | 20.46 |
|  |  | 30 - 31 | 74.96 | 85.25 | 17.54 |
|  | Rapid Time Movement Ratio (%) | 0 - 1 | 15.14 | 12.70 | 13.31 |
|  |  | 5 - 6 | 11.87 | 10.10 | 7.82 |
|  |  | 10 - 11 | 9.64 | 7.45 | 9.21 |
|  |  | 15 - 16 | 8.47 | 6.45 | 9.96 |
|  |  | 20 - 21 | 7.67 | 3.95 | 9.29 |
|  |  | 25 – 26 | 5.53 | 5.00 | 4.88 |
|  |  | 30 - 31 | 8.23 | 4.55 | 11.73 |
|  | Time in Top Duration (%) | 0 - 1 | 0.20 | 0.00 | 0.94 |
|  |  | 5 - 6 | 8.75 | 6.13 | 10.56 |
|  |  | 10 - 11 | 11.07 | 6.85 | 13.73 |
|  |  | 15 - 16 | 9.58 | 1.57 | 15.95 |
|  |  | 20 - 21 | 7.16 | 0.29 | 12.80 |
|  |  | 25 – 26 | 12.01 | 5.32 | 15.24 |
|  |  | 30 - 31 | 11.54 | 0.67 | 16.98 |
|  | Number of Entries to The Top | 0 - 1 | 0.17 | 0.00 | 0.65 |
|  |  | 5 - 6 | 3.70 | 3.00 | 3.83 |
|  |  | 10 - 11 | 2.80 | 1.00 | 3.21 |
|  |  | 15 - 16 | 2.20 | 1.00 | 3.10 |
|  |  | 20 - 21 | 1.63 | 0.50 | 2.39 |
|  |  | 25 – 26 | 3.03 | 2.00 | 3.54 |
|  |  | 30 - 31 | 2.33 | 0.50 | 4.09 |
|  | Latency to Enter the Top (s) | 0 - 1 | 57.43 | 60.00 | 10.43 |
|  |  | 5 - 6 | 28.69 | 23.16 | 25.44 |
|  |  | 10 - 11 | 27.51 | 12.01 | 27.55 |
|  |  | 15 - 16 | 36.41 | 37.32 | 23.42 |
|  |  | 20 - 21 | 42.02 | 59.75 | 23.01 |
|  |  | 25 – 26 | 26.40 | 13.32 | 26.81 |
|  |  | 30 - 31 | 32.08 | 38.30 | 28.67 |
|  | Total Distance Traveled In The Top (cm) | 0 - 1 | 0.99 | 0.00 | 4.26 |
|  |  | 5 - 6 | 40.86 | 25.44 | 45.71 |
|  |  | 10 - 11 | 42.69 | 25.16 | 49.33 |
|  |  | 15 - 16 | 32.93 | 7.92 | 50.72 |
|  |  | 20 - 21 | 24.79 | 0.83 | 46.66 |
|  |  | 25 – 26 | 41.26 | 21.41 | 48.45 |
|  |  | 30 - 31 | 38.65 | 2.21 | 56.04 |
|  | Thigmotaxis (cm) | 0 - 1 | 5.40 | 4.83 | 1.84 |
|  |  | 5 - 6 | 5.56 | 5.61 | 1.09 |
|  |  | 10 - 11 | 5.71 | 5.87 | 1.10 |
|  |  | 15 - 16 | 5.58 | 5.40 | 1.42 |
|  |  | 20 - 21 | 5.36 | 5.07 | 1.38 |
|  |  | 25 – 26 | 5.85 | 5.55 | 1.45 |
|  |  | 30 - 31 | 5.42 | 5.43 | 1.14 |
| **WIK Zebrafish** | Average Speed (cm/s) | 0 - 1 | 2.66 | 2.63 | 2.28 |
|  |  | 5 - 6 | 4.81 | 5.37 | 2.13 |
|  |  | 10 - 11 | 5.09 | 4.82 | 1.29 |
|  |  | 15 - 16 | 5.12 | 5.16 | 1.27 |
|  |  | 20 - 21 | 4.86 | 4.83 | 1.91 |
|  |  | 25 – 26 | 4.73 | 4.56 | 2.01 |
|  |  | 30 - 31 | 4.63 | 5.16 | 1.60 |
|  | Freezing Time Movement Ratio (%) | 0 - 1 | 51.83 | 56.80 | 40.06 |
|  |  | 5 - 6 | 15.80 | 5.70 | 23.74 |
|  |  | 10 - 11 | 8.27 | 7.10 | 5.04 |
|  |  | 15 - 16 | 6.73 | 5.30 | 5.05 |
|  |  | 20 - 21 | 14.17 | 6.90 | 20.86 |
|  |  | 25 – 26 | 15.41 | 8.80 | 14.55 |
|  |  | 30 - 31 | 11.09 | 5.60 | 12.25 |
|  | Swimming Time Movement Ratio (%) | 0 - 1 | 43.58 | 33.00 | 36.58 |
|  |  | 5 - 6 | 75.18 | 81.10 | 20.97 |
|  |  | 10 - 11 | 83.13 | 85.00 | 9.40 |
|  |  | 15 - 16 | 85.54 | 89.20 | 7.31 |
|  |  | 20 - 21 | 77.48 | 81.90 | 20.57 |
|  |  | 25 – 26 | 75.65 | 76.70 | 11.56 |
|  |  | 30 - 31 | 82.70 | 87.10 | 10.89 |
|  | Rapid Time Movement Ratio (%) | 0 - 1 | 4.55 | 3.70 | 5.02 |
|  |  | 5 - 6 | 8.99 | 6.00 | 9.12 |
|  |  | 10 - 11 | 8.57 | 6.20 | 7.56 |
|  |  | 15 - 16 | 7.69 | 5.90 | 7.14 |
|  |  | 20 - 21 | 8.31 | 4.20 | 8.37 |
|  |  | 25 – 26 | 8.91 | 5.90 | 8.19 |
|  |  | 30 - 31 | 6.19 | 5.20 | 5.79 |
|  | Time in Top Duration (%) | 0 - 1 | 3.45 | 0.00 | 8.92 |
|  |  | 5 - 6 | 19.00 | 13.10 | 21.29 |
|  |  | 10 - 11 | 27.30 | 26.57 | 20.43 |
|  |  | 15 - 16 | 36.73 | 27.17 | 27.08 |
|  |  | 20 - 21 | 42.17 | 48.07 | 19.15 |
|  |  | 25 – 26 | 32.33 | 30.60 | 26.67 |
|  |  | 30 - 31 | 42.03 | 46.30 | 26.85 |
|  | Number of Entries to The Top | 0 - 1 | 1.30 | 0.00 | 2.74 |
|  |  | 5 - 6 | 4.17 | 4.00 | 4.64 |
|  |  | 10 - 11 | 6.70 | 6.00 | 4.42 |
|  |  | 15 - 16 | 8.00 | 7.00 | 5.62 |
|  |  | 20 - 21 | 8.48 | 6.00 | 6.78 |
|  |  | 25 – 26 | 6.87 | 6.00 | 5.67 |
|  |  | 30 - 31 | 7.43 | 7.00 | 5.41 |
|  | Latency to Enter the Top (s) | 0 - 1 | 45.59 | 60.00 | 22.92 |
|  |  | 5 - 6 | 25.95 | 8.88 | 28.14 |
|  |  | 10 - 11 | 10.62 | 5.18 | 16.43 |
|  |  | 15 - 16 | 6.58 | 0.20 | 15.87 |
|  |  | 20 - 21 | 7.33 | 0.02 | 15.06 |
|  |  | 25 – 26 | 8.61 | 2.30 | 17.02 |
|  |  | 30 - 31 | 9.52 | 0.54 | 20.26 |
|  | Total Distance Traveled In The Top (cm) | 0 - 1 | 10.86 | 0.00 | 29.27 |
|  |  | 5 - 6 | 75.21 | 63.04 | 87.13 |
|  |  | 10 - 11 | 95.51 | 92.45 | 67.29 |
|  |  | 15 - 16 | 130.78 | 85.02 | 109.16 |
|  |  | 20 - 21 | 140.67 | 147.37 | 78.45 |
|  |  | 25 – 26 | 100.67 | 89.48 | 75.65 |
|  |  | 30 - 31 | 125.73 | 143.72 | 78.17 |
|  | Thigmotaxis (cm) | 0 - 1 | 5.65 | 4.06 | 7.86 |
|  |  | 5 - 6 | 5.53 | 4.91 | 1.87 |
|  |  | 10 - 11 | 6.58 | 6.34 | 2.01 |
|  |  | 15 - 16 | 6.58 | 6.69 | 1.98 |
|  |  | 20 - 21 | 6.36 | 6.34 | 1.91 |
|  |  | 25 – 26 | 6.46 | 6.45 | 2.16 |
|  |  | 30 - 31 | 6.77 | 6.80 | 2.05 |
