# Supplementary material for: Which Zebrafish Strains Are More Suitable to Perform Behavioral Studies? A Comprehensive Comparison by Phenomic Approach
Source: Biology (Basel). 2020 Aug 1;9(8):200. doi: 10.3390/biology9080200 (PMC7465594; doi:10.3390/biology9080200)
Supplement: Supplementary file 1 [file biology-09-00200-s001.zip › Table S3.docx]

**Table S3.** The mean, median, and SD of *Puntigrus tetrazona* behavior endpoints in each behavioral test.

| Zebrafish Line | Novel Tank Test Endpoint | Time Interval (min) | Mean | Median | SD |
| --- | --- | --- | --- | --- | --- |
| **Novel Tank Test** | Average Speed (cm/s) | 0 - 1 | 3.59 | 3.42 | 0.93 |
|  |  | 5 - 6 | 3.77 | 3.44 | 1.19 |
|  |  | 10 - 11 | 3.44 | 3.59 | 0.96 |
|  |  | 15 - 16 | 3.51 | 3.35 | 0.83 |
|  |  | 20 - 21 | 3.49 | 3.45 | 0.94 |
|  |  | 25 – 26 | 3.38 | 3.40 | 0.99 |
|  |  | 30 - 31 | 3.45 | 3.70 | 0.94 |
|  | Freezing Time Movement Ratio (%) | 0 - 1 | 10.13 | 9.20 | 4.95 |
|  |  | 5 - 6 | 10.50 | 10.90 | 5.19 |
|  |  | 10 - 11 | 11.88 | 9.90 | 7.55 |
|  |  | 15 - 16 | 11.00 | 10.30 | 5.00 |
|  |  | 20 - 21 | 11.91 | 9.80 | 7.44 |
|  |  | 25 – 26 | 13.21 | 11.40 | 8.07 |
|  |  | 30 - 31 | 13.13 | 10.10 | 11.65 |
|  | Swimming Time Movement Ratio (%) | 0 - 1 | 87.75 | 89.00 | 4.14 |
|  |  | 5 - 6 | 86.20 | 87.10 | 3.83 |
|  |  | 10 - 11 | 86.03 | 86.20 | 6.80 |
|  |  | 15 - 16 | 86.92 | 88.10 | 4.16 |
|  |  | 20 - 21 | 85.91 | 87.60 | 6.80 |
|  |  | 25 – 26 | 84.95 | 87.30 | 7.11 |
|  |  | 30 - 31 | 84.94 | 87.10 | 10.97 |
|  | Rapid Time Movement Ratio (%) | 0 - 1 | 2.10 | 1.40 | 2.18 |
|  |  | 5 - 6 | 3.28 | 1.30 | 4.64 |
|  |  | 10 - 11 | 2.05 | 1.20 | 2.91 |
|  |  | 15 - 16 | 2.06 | 1.30 | 2.62 |
|  |  | 20 - 21 | 2.14 | 1.20 | 2.85 |
|  |  | 25 – 26 | 1.80 | 1.00 | 2.07 |
|  |  | 30 - 31 | 1.90 | 1.20 | 2.15 |
|  | Time in Top Duration (%) | 0 - 1 | 38.03 | 29.37 | 27.97 |
|  |  | 5 - 6 | 39.15 | 37.20 | 22.89 |
|  |  | 10 - 11 | 34.21 | 34.97 | 24.02 |
|  |  | 15 - 16 | 37.30 | 34.70 | 25.70 |
|  |  | 20 - 21 | 41.82 | 39.73 | 25.64 |
|  |  | 25 – 26 | 37.65 | 37.67 | 25.19 |
|  |  | 30 - 31 | 35.27 | 33.93 | 25.01 |
|  | Number of Entries to The Top | 0 - 1 | 10.21 | 10.00 | 5.34 |
|  |  | 5 - 6 | 10.17 | 10.00 | 5.17 |
|  |  | 10 - 11 | 9.48 | 8.00 | 5.43 |
|  |  | 15 - 16 | 7.93 | 8.00 | 3.66 |
|  |  | 20 - 21 | 9.45 | 9.00 | 4.79 |
|  |  | 25 – 26 | 8.97 | 9.00 | 5.60 |
|  |  | 30 - 31 | 9.03 | 7.00 | 6.29 |
|  | Latency to Enter the Top (s) | 0 - 1 | 4.59 | 1.20 | 8.88 |
|  |  | 5 - 6 | 5.71 | 2.18 | 11.70 |
|  |  | 10 - 11 | 4.09 | 1.12 | 11.16 |
|  |  | 15 - 16 | 7.58 | 1.80 | 12.98 |
|  |  | 20 - 21 | 7.46 | 1.02 | 15.82 |
|  |  | 25 – 26 | 10.63 | 1.40 | 18.87 |
|  |  | 30 - 31 | 8.65 | 1.22 | 15.82 |
|  | Total Distance Traveled In The Top (cm) | 0 - 1 | 86.66 | 63.68 | 65.38 |
|  |  | 5 - 6 | 105.27 | 94.46 | 79.04 |
|  |  | 10 - 11 | 86.50 | 78.27 | 70.78 |
|  |  | 15 - 16 | 87.75 | 73.49 | 61.49 |
|  |  | 20 - 21 | 99.13 | 84.45 | 71.36 |
|  |  | 25 – 26 | 88.35 | 77.62 | 64.54 |
|  |  | 30 - 31 | 82.48 | 69.88 | 59.45 |
|  | Thigmotaxis (cm) | 0 - 1 | 6.15 | 6.32 | 2.26 |
|  |  | 5 - 6 | 6.01 | 6.16 | 1.88 |
|  |  | 10 - 11 | 6.39 | 6.23 | 1.86 |
|  |  | 15 - 16 | 6.27 | 5.63 | 1.77 |
|  |  | 20 - 21 | 7.17 | 6.62 | 2.11 |
|  |  | 25 – 26 | 6.17 | 5.76 | 1.96 |
|  |  | 30 - 31 | 6.69 | 6.96 | 2.14 |
| **Mirror Biting Test** | Mirror Biting Time Percentage (%) | 0-5 | 54.58 | 55.09 | 30.77 |
|  | Longest Duration in The Mirror Side (%) | 0-5 | 23.34 | 18.31 | 21.60 |
| **Predator Avoidance Test** | Approaching Predator Time Percentage (%) | 0-5 | 12.57 | 7.46 | 11.74 |
|  | Average Distance to the Predator’s Separator (cm) | 0-5 | 7.77 | 7.86 | 1.56 |
| **Social Interaction Test** | Conspecific Interaction Time Percentage (%) | 0-5 | 67.49 | 68.39 | 19.04 |
|  | Longest Conspecific Interaction Percentage (%) | 0-5 | 18.67 | 12.50 | 12.95 |
|  | Average Distance to the Conspecific’s Separator (cm) | 0-5 | 3.05 | 2.82 | 1.06 |
| **Shoaling Test** | Average Inter-fish Distance (cm) | 0-5 | 4.25 | 3.95 | 0.99 |
|  | Average Shoal Area (cm^2^) | 0-5 | 4.00 | 4.04 | 0.78 |
|  | Average Nearest Neighbor Distance (cm) | 0-5 | 3.10 | 2.96 | 0.60 |
|  | Average Farthest Neighbor Distance (cm) | 0-5 | 5.40 | 5.12 | 1.10 |
